# Supplementary material for: Correlation-based tests for the formal comparison of polygenic scores in multiple populations
Source: PLoS Genet. 2024 Apr 26;20(4):e1011249. doi: 10.1371/journal.pgen.1011249 (PMC11078427; doi:10.1371/journal.pgen.1011249)
Supplement: S1 Appendix — (PDF) [file pgen.1011249.s001.pdf]

S1 Appendix: Supplemental Methods

A Deriving Elements of Covariance Matrix for Vector of Sample Correlations

To compute the asymptotic variance-covariance matrix of the sample correlation statistics for a single population j, $\hat{\Sigma}_\infty(u_j)$, where $u_j = (r_{Y,1,j}, r_{Y,2,j}, ...r_{Y,P,j})$, and $r_{Y,i,j}$ is the sample correlation of the ith PGS with the outcome Y in the jth population,

$$\hat{\Sigma}_\infty(u_j) = \begin{pmatrix} var(r_{Y,1,j}) & cov(r_{Y,1,j}, r_{Y,2,j}) & \dots & cov(r_{Y,1,j}, r_{Y,P,j}) \\ cov(r_{Y,1,j}, r_{Y,2,j}) & var(r_{Y,2,j}) & \dots & cov(r_{Y,2,j}, r_{Y,P,j}) \\ \vdots & \vdots & \ddots & \vdots \\ cov(r_{Y,1,j}, r_{Y,P,j}) & \dots & \dots & var(r_{Y,P,j}) \end{pmatrix}$$

we use the following expressions from Olkin and Finn, 1990:
the variance of sample correlation $r_{Y,i,j}$,:

$$var(r_{Y,i,j}) = (1 - r^2_{Y,i,j})^2/n$$

the covariance of two correlations $r_{Y,i,j}$ and $r_{Y,m,j}$:

$$cov(r_{Y,i,j}, r_{Y,m,j}) = [1/2(2r_{i,m,j} - r_{Y,i,j}r_{Y,m,j})(1 - r^2_{Y,i,j} - r^2_{Y,m,j} - r^2_{i,m,j}) + r^3_{i,m,j}]/n$$

To define the variance-covariance matrix of the sample correlation statistics when comparing sample correlations from K populations parametrically according to Olkin and Finn, let $u = (u_1, u_2, ..., u_K)$. Then, $\hat{\Sigma}_\infty(u)$ is a block matrix made up of the population-specific variance-covariance matrices:

$$\hat{\Sigma}_\infty(u) = \begin{pmatrix} \hat{\Sigma}_\infty(u_1) & 0 & \dots & 0 \\ 0 & \hat{\Sigma}_\infty(u_2) & \dots & 0 \\ \vdots & \vdots & \ddots & \vdots \\ 0 & 0 & \dots & \hat{\Sigma}_\infty(u_K) \end{pmatrix}$$

B Contrast Matrices for Coranova Hypotheses

The contrast matrices for each of the three Coranova hypotheses from Bilker et al can be derived using the following expressions, where k is number of groups and p is number of polygenic scores (in Bilker et al. p = number of scores + 1).

Between Hypothesis:

$$C_B = \mathbf{1'}_{k-1} \otimes \mathbf{1}_p \mid \mathbf{I}_{k-1} \otimes -\mathbf{1}_p$$

Within Hypothesis:

$$C_W = \mathbf{1}_k \otimes (\mathbf{1'}_{p-1} \mid -\mathbf{I}_{p-1})$$

Interaction Hypothesis:

$$C_I = \mathbf{1'}_{k-1} \otimes (\mathbf{1}_{p-1} \mid \mathbf{I}_{p-1}) \mid \mathbf{1}_{k-1} \otimes (-\mathbf{1'}_{p-1} \mid \mathbf{I'}_{p-1})$$

C Test Statistics for Coranova Hypotheses

Following Eq 2, using the contrast matrices from section B, the test statistics for each three Coranova hypotheses can be defined with the following:

Between Hypothesis:

$$S_B = (\mathbf{C_B}\hat{\mathbf{u}})'(\mathbf{C_B}\hat{\mathbf{\Sigma}}(\mathbf{u})\mathbf{C'_B})^{-1}(\mathbf{C_B}\hat{\mathbf{u}}) \sim \chi^2_{k-1}$$

Within Hypothesis:

$$S_W = (\mathbf{C_W}\hat{\mathbf{u}})'(\mathbf{C_W}\hat{\mathbf{\Sigma}}(\mathbf{u})\mathbf{C'_W})^{-1}(\mathbf{C_W}\hat{\mathbf{u}}) \sim \chi^2_{p-1}$$

Interaction Hypothesis:

$$S_I = (\mathbf{C_I}\hat{\mathbf{u}})'(\mathbf{C_I}\hat{\mathbf{\Sigma}}(\mathbf{u})\mathbf{C'_I})^{-1}(\mathbf{C_I}\hat{\mathbf{u}}) \sim \chi^2_{(p-1)(k-1)}$$

D Simulations of Binary Traits

To simulate binary trait data, we adapted our continuous trait simulations by first simulating a continuous outcome Y and PGS according to the procedure used for continuous traits and then converting the continuous outcome Y to binary by assigning the upper 10% of the distribution to be cases, and the remaining distribution to be controls, generating a binary outcome with prevalence of 10%. We then applied the

nonparametric implementation of Coranova available in the function `perform_coranova_nonparametric` with 1000 bootstrap samples and 1000 permutations.

E Contrast Matrices for Additional Hypothesis Tests

LDL Cholesterol example:
Comparing scores built with Pruning and Thresholding to the scores built with PRS-CS

Let $r_{i,j,k}$ be the correlation between PGS i and LDL cholesterol in 1KG genetic-similarity group j and method k. Then, the vector u is:

$u = (r_{MULTI,PT,afr}, r_{AFR,PT,afr}, r_{EUR,PT,afr}, r_{HIS,PT,afr}, r_{EAS,PT,afr}, r_{SAS,PT,afr},$
$r_{MULTI,PRS-CS,afr}, r_{AFR,PRS-CS,afr}, r_{EUR,PRS-CS,afr}, r_{HIS,PRS-CS,afr}, r_{EAS,PRS-CS,afr}, r_{SAS,PRS-CS,afr},$
$r_{MULTI,PT,eur}, r_{AFR,PT,eur}, r_{EUR,PT,eur}, r_{HIS,PT,eur}, r_{EAS,PT,eur}, r_{SAS,PT,eur},$
$r_{MULTI,PRS-CS,eur}, r_{AFR,PRS-CS,eur}, r_{EUR,PRS-CS,eur}, r_{HIS,PRS-CS,eur}, r_{EAS,PRS-CS,eur}, r_{SAS,PRS-CS,eur},$
$r_{MULTI,PT,amr}, r_{AFR,PT,amr}, r_{EUR,PT,amr}, r_{HIS,PT,amr}, r_{EAS,PT,amr}, r_{SAS,PT,amr},$
$r_{MULTI,PRS-CS,amr}, r_{AFR,PRS-CS,amr}, r_{EUR,PRS-CS,amr}, r_{HIS,PRS-CS,amr}, r_{EAS,PRS-CS,amr}, r_{SAS,PRS-CS,amr})$

To devise a hypothesis test in which we compare the PT PGS to the PRS-CS PGS,

let $A = \begin{pmatrix} 1 & 1 & 1 \end{pmatrix} \otimes \begin{pmatrix} 1 & 0 & 0 & 0 & 0 & 0 & -1 & 0 & 0 & 0 & 0 & 0 \\ 0 & 1 & 0 & 0 & 0 & 0 & 0 & -1 & 0 & 0 & 0 & 0 \\ 0 & 0 & 1 & 0 & 0 & 0 & 0 & 0 & -1 & 0 & 0 & 0 \\ 0 & 0 & 0 & 1 & 0 & 0 & 0 & 0 & 0 & -1 & 0 & 0 \\ 0 & 0 & 0 & 0 & 1 & 0 & 0 & 0 & 0 & 0 & -1 & 0 \\ 0 & 0 & 0 & 0 & 0 & 1 & 0 & 0 & 0 & 0 & 0 & -1 \end{pmatrix}$

Then we can define our null hypothesis to be

$H_0 : uA =$

$$\begin{pmatrix} (r_{MULT,afr,PT} + r_{MULT,amr,PT} + r_{MULT,eur,PT}) - (r_{MULT,afr,PCS} + r_{MULT,amr,PCS} + r_{MULT,eur,PCS}) \\ (r_{AFR,afr,PT} + r_{AFR,amr,PT} + r_{AFR,eur,PT}) - (r_{AFR,afr,PCS} + r_{AFR,amr,PCS} + r_{AFR,eur,PCS}) \\ (r_{HIS,afr,PT} + r_{HIS,amr,PT} + r_{HIS,eur,PT}) - (r_{HIS,afr,PCS} + r_{HIS,amr,PCS} + r_{HIS,eur,PCS}) \\ (r_{EUR,afr,PT} + r_{EUR,amr,PT} + r_{EUR,eur,PT}) - (r_{EUR,afr,PCS} + r_{EUR,amr,PCS} + r_{EUR,eur,PCS}) \\ (r_{EAS,afr,PT} + r_{EAS,amr,PT} + r_{EAS,eur,PT}) - (r_{EAS,afr,PCS} + r_{EAS,amr,PCS} + r_{EAS,eur,PCS}) \\ (r_{SAS,afr,PT} + r_{SAS,amr,PT} + r_{SAS,eur,PT}) - (r_{SAS,afr,PCS} + r_{SAS,amr,PCS} + r_{SAS,eur,PCS}) \end{pmatrix} = \begin{pmatrix} 0 \\ 0 \\ 0 \\ 0 \\ 0 \\ 0 \end{pmatrix}$$

Comparing multi-ancestry PGS to ancestry-specific PGS

Let $r_{i,j}$ be the correlation between PT PGS i and LDL cholesterol in 1KG genetic-similarity group j.

Then, the vector u is:

$u = (r_{MULTI,afr}, r_{AFR,afr}, r_{EUR,afr}, r_{HIS,afr}, r_{EAS,afr}, r_{SAS,afr},$
$r_{MULTI,eur}, r_{AFR,eur}, r_{EUR,eur}, r_{HIS,eur}, r_{EAS,eur}, r_{SAS,eur},$
$r_{MULTI,amr}, r_{AFR,amr}, r_{EUR,amr}, r_{HIS,amr}, r_{EAS,amr}, r_{SAS,amr})$

To devise a hypothesis test in which we compare the multi-ancestry PGS to the ancestry-specific PGS,

let $A = \begin{pmatrix} 1 & -1 & 0 & 0 & 0 & 0 & 0 & 0 & 0 & 0 & 0 & 0 & 0 & 0 & 0 & 0 & 0 & 0 \\ 0 & 0 & 0 & 0 & 0 & 0 & 1 & 0 & -1 & 0 & 0 & 0 & 0 & 0 & 0 & 0 & 0 & 0 \\ 0 & 0 & 0 & 0 & 0 & 0 & 0 & 0 & 0 & 0 & 0 & 0 & 1 & 0 & 0 & -1 & 0 & 0 \end{pmatrix}$

Then we can define our null hypothesis to be

$H_0 : uA = \begin{pmatrix} r_{MULTI,afr} - r_{AFR,afr} \\ r_{MULTI,eur} - r_{EUR,eur} \\ r_{MULTI,amr} - r_{HIS,amr} \end{pmatrix} = \begin{pmatrix} 0 \\ 0 \\ 0 \end{pmatrix}$
