# Supplementary material for: Correlation-based tests for the formal comparison of polygenic scores in multiple populations
Source: PLoS Genet. 2024 Apr 26;20(4):e1011249. doi: 10.1371/journal.pgen.1011249 (PMC11078427; doi:10.1371/journal.pgen.1011249)
Supplement: S2 Appendix — (PDF) [file pgen.1011249.s002.pdf]

S2 Appendix: Supplemental Results

A Power as Function of Parameters

We observe that for the three Coranova hypotheses, power increases as the scores' correlation with the outcome increases. We also observe that for the between test, holding all else constant, as the correlation between the scores increases, the power to detect a difference in score performance across groups decreases, while for the within and interaction tests, the reverse is observed and the power to detect a difference in score performance, and a difference in pattern of score performance, respectively, increases as the correlation between the scores increases. These observations can be understood by examining the test-statistics themselves.

Let's consider the simple case where we want to compare two polygenic scores evaluated in two distinct population samples. This will allow us to write out our test statistics in terms of the variance and covariance of the correlation values (r_{ij}s), and understand how the test-statistics change according to these parameters.

Let r_{ij} be the correlation of the ith polygenic score with outcome Y in the jth population. Thus, in our setting we have r_{11}, r_{21}, r_{21} and r_{22}. To compare the performance of the scores within the groups we use the within test with the following test-statistic:

$$\chi^2_{within} = \frac{(r_{11} + r_{12} - (r_{21} + r_{22}))^2}{var(r_{11}) - 2cov(r_{11}, r_{21}) + var(r_{12}) + var(r_{21}) - 2cov(r_{12}, r_{22}) + var(r_{22})}$$

To compare the performance of the scores across the groups we use the between test with the following test-statistic:

$$\chi^2_{between} = \frac{(r_{11} + r_{21} - (r_{12} + r_{22}))^2}{var(r_{11}) + 2cov(r_{11}, r_{21}) + var(r_{12}) + var(r_{21}) + 2cov(r_{12}, r_{22}) + var(r_{22})}$$

To compare the pattern of score performance we use the interaction test with the following test-statistic:

$$\chi^2_{interaction} = \frac{(r_{11} - r_{12} - r_{21} + r_{22})^2}{var(r_{11}) - 2cov(r_{11}, r_{21}) + var(r_{12}) + var(r_{21}) - 2cov(r_{12}, r_{22}) + var(r_{22})}$$

As referenced in S1 Appendix Supplemental Methods section A, where $r_{Y,i,j}$ is the sample correlation of the ith PGS with the outcome Y in the jth population,
the variance of sample correlation $r_{Y,i,j}$:

$var(r_{Y,i,j}) = (1 - r^2_{Y,i,j})^2/n$

the covariance of two correlations $r_{Y,i,j}$ and $r_{Y,m,j}$:

$cov(r_{Y,i,j}, r_{Y,m,j}) = [1/2(2r_{i,m,j} - r_{Y,i,j}r_{Y,m,j})(1 - r^2_{Y,i,j} - r^2_{Y,m,j} - r^2_{i,m,j}) + r^3_{i,m,j}]/n$

Observations:
To understand these observations, we can consider the denominators of the test statistics.

1. **For all three tests, power increases as scores' correlation with outcome increases.** Within all three denominators, there are multiple positive variance terms. As r_{ij}, the correlation between the score and outcome, increases between 0 and 1, the variance $var(r_{ij})$ decreases. Thus, holding all else constant, as the correlation of the scores with the outcome increase in the sample, all three test-statistics increase and power to detect a significant difference increases.

2. **For the between test, power decreases as the correlation between the scores increase.** Looking at the denominator, we can see the covariance terms are positive. Thus, as the correlation between the scores increases and the covariance terms increase, the overall test-statistic will decrease. Thus, the power of the test decreases.

3. **For the within and interaction tests, power increases as the correlation between the scores increase.** Unlike the between test, the within and interaction tests' denominators have negative covariance terms. Thus, as the correlation between the scores increases and the covariance terms increase, the overall test-statistic will also increase and the power of the test increases as well.

B Comparison to R^2 Redux

To compare our correlation-based method to R2Redux, we performed simulations to assess the type 1 error rate and power of both methods when assessing the difference in performance between two polygenic scores in a single population. We find that our correlation-based method has well controlled type I error in all simulation settings (S3 Appendix Supplemental Figures Fig I). With sample sizes of 1000 individuals, we also find our correlation-based method has equal or higher power than R2Redux (S3 Appendix Supplemental Figures Fig J). When we compare its performance to R2Redux, both methods have well-controlled type I error and equal power when the correlation between the PGS and outcome is high (high τ). When the correlation between the PGS and outcome is low (low τ), the correlation-based tests are more powerful than the tests implemented in R2Redux. However, with larger sample size of 10000, the methods perform nearly identically with respect to type 1 error rate and power (S3 Appendix Supplemental Figures Figs K and L).

C Assessment of Nonparametric Implementation of Coranova Applied to Binary Traits

We find that with 1000 bootstrap samples, and 1000 permutations, the nonparametric implementations of Coranova have well controlled type 1 error when applied to 3 polygenic scores in 2 population samples of size n = 1000 (S3 Appendix Supplemental Figures Fig M). Compared to the parametric implementation of Coranova applied to continuous outcomes of equal sample size, we have diminished power to reject the three Coranova hypotheses when applied to binary data with a prevalence of 0.1 (S3 Appendix Supplemental Figures Fig N).
