# Supplementary material for: Correlation-based tests for the formal comparison of polygenic scores in multiple populations
Source: PLoS Genet. 2024 Apr 26;20(4):e1011249. doi: 10.1371/journal.pgen.1011249 (PMC11078427; doi:10.1371/journal.pgen.1011249)
Supplement: S3 Appendix — (PDF) [file pgen.1011249.s003.pdf]

S3 Appendix: Supplemental Figures

A Simulation Results Assessing Coranova Hypothesis Tests

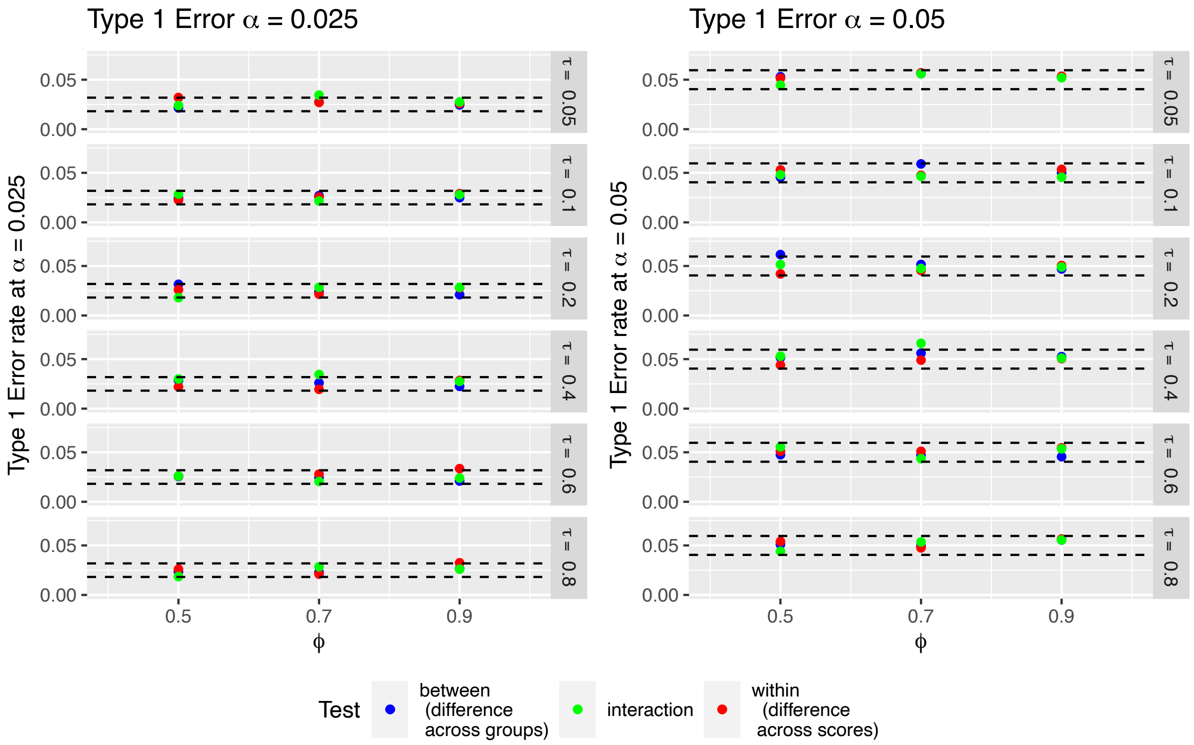

Supplemental Figure A: Type I error of Parametric Implementation of Coranova with two populations, each of sample size 500. Each point represents the proportion of tests in 1000 simulations with parameters ϕ, τ and δ in which the null hypothesis was rejected at specified alpha level. Dashed lines indicate 95% confidence interval for specified alpha given sample size.

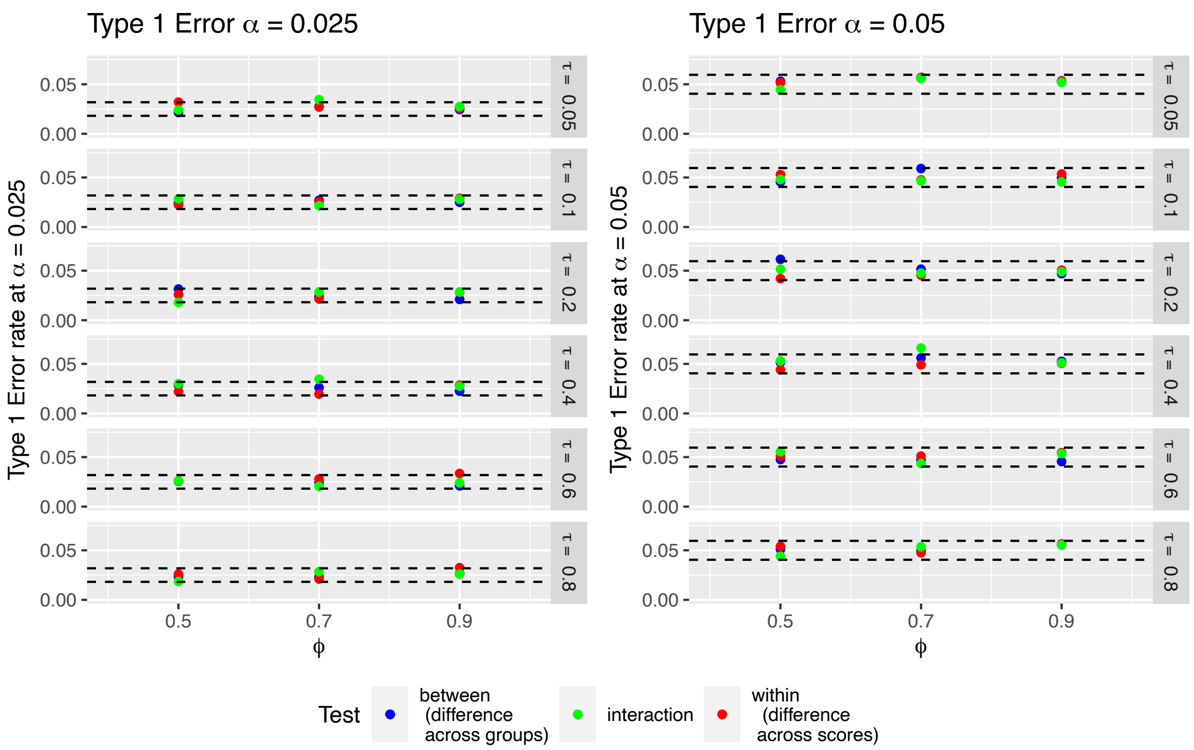

Supplemental Figure B: Type I error of Parametric Implementation of Coranova with two populations, each of sample size 5000. Each point represents the proportion of tests in 1000 simulations with parameters ϕ, τ and δ in which the null hypothesis was rejected at specified alpha level. Dashed lines indicate 95% confidence interval for specified alpha given sample size.

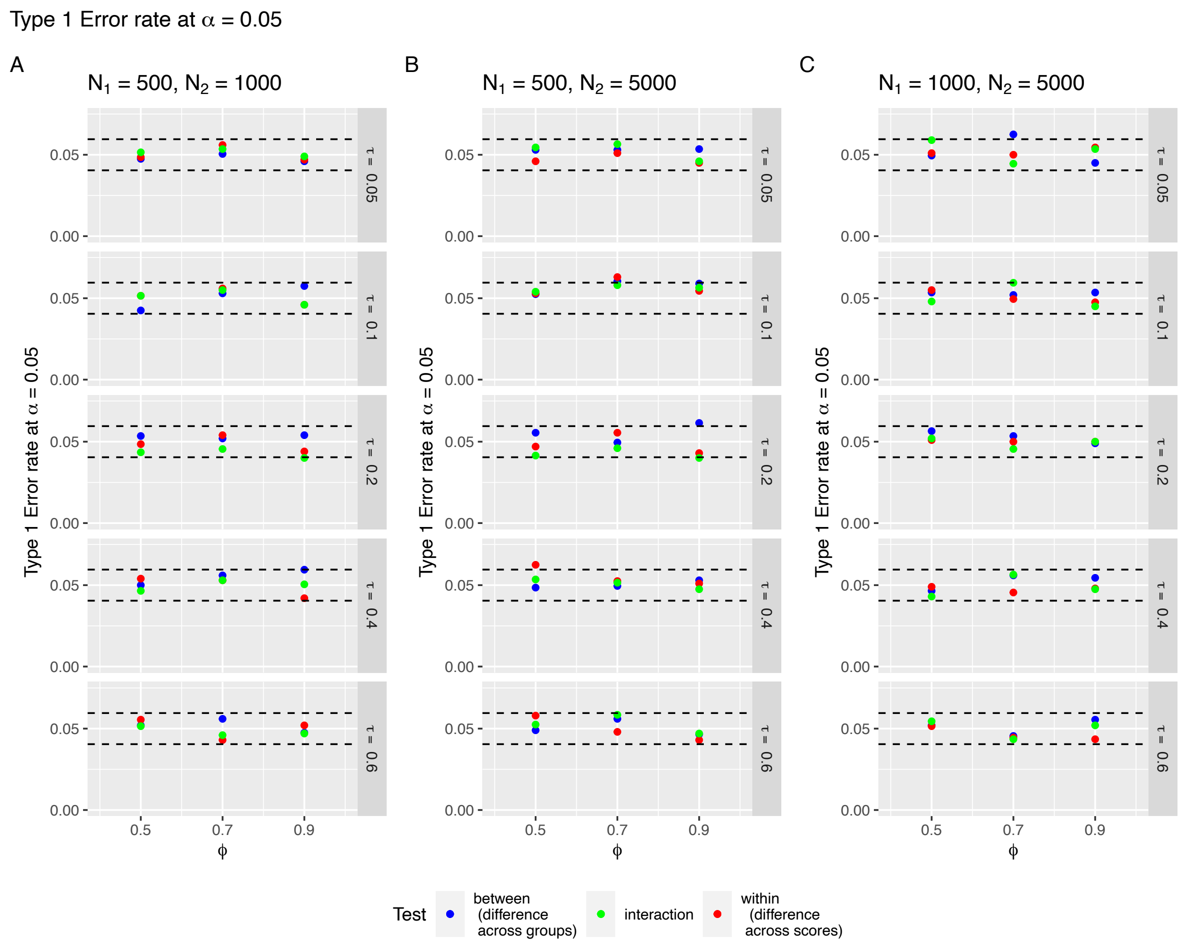

Supplemental Figure C: Type I error of Parametric Implementation of Coranova with two populations, of varying sample sizes. Each point represents the proportion of tests in 1000 simulations with parameters ϕ, τ and δ in which the null hypothesis was rejected at specified alpha level. Dashed lines indicate 95% confidence interval for specified alpha given sample size.

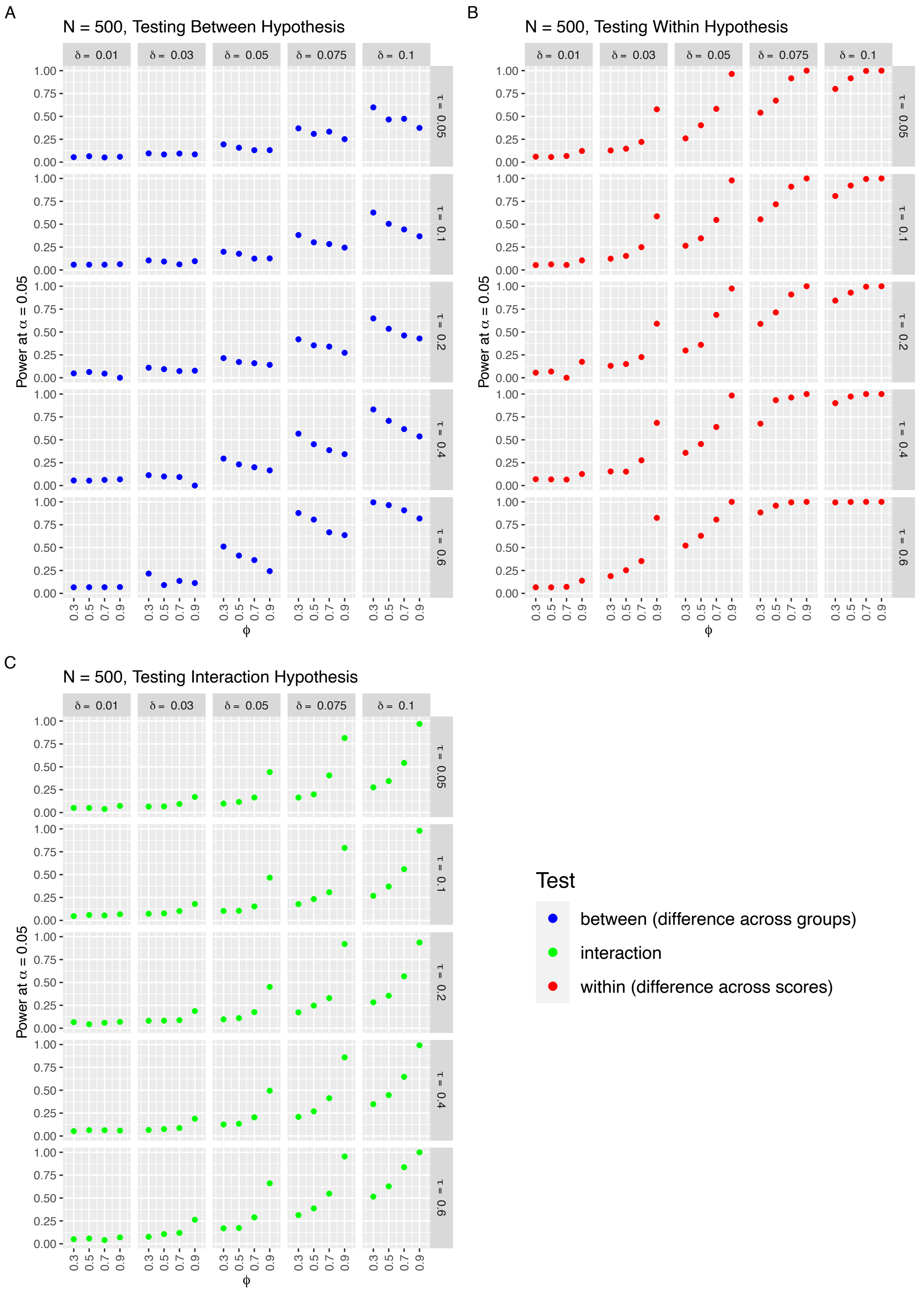

Supplemental Figure D: Power of Parametric Implementation of Coranova with two populations, each of sample size 500. Each point represents the proportion of tests in 1000 simulations with parameters ϕ, τ and δ in which the null hypothesis was rejected at significance level alpha = 0.05.

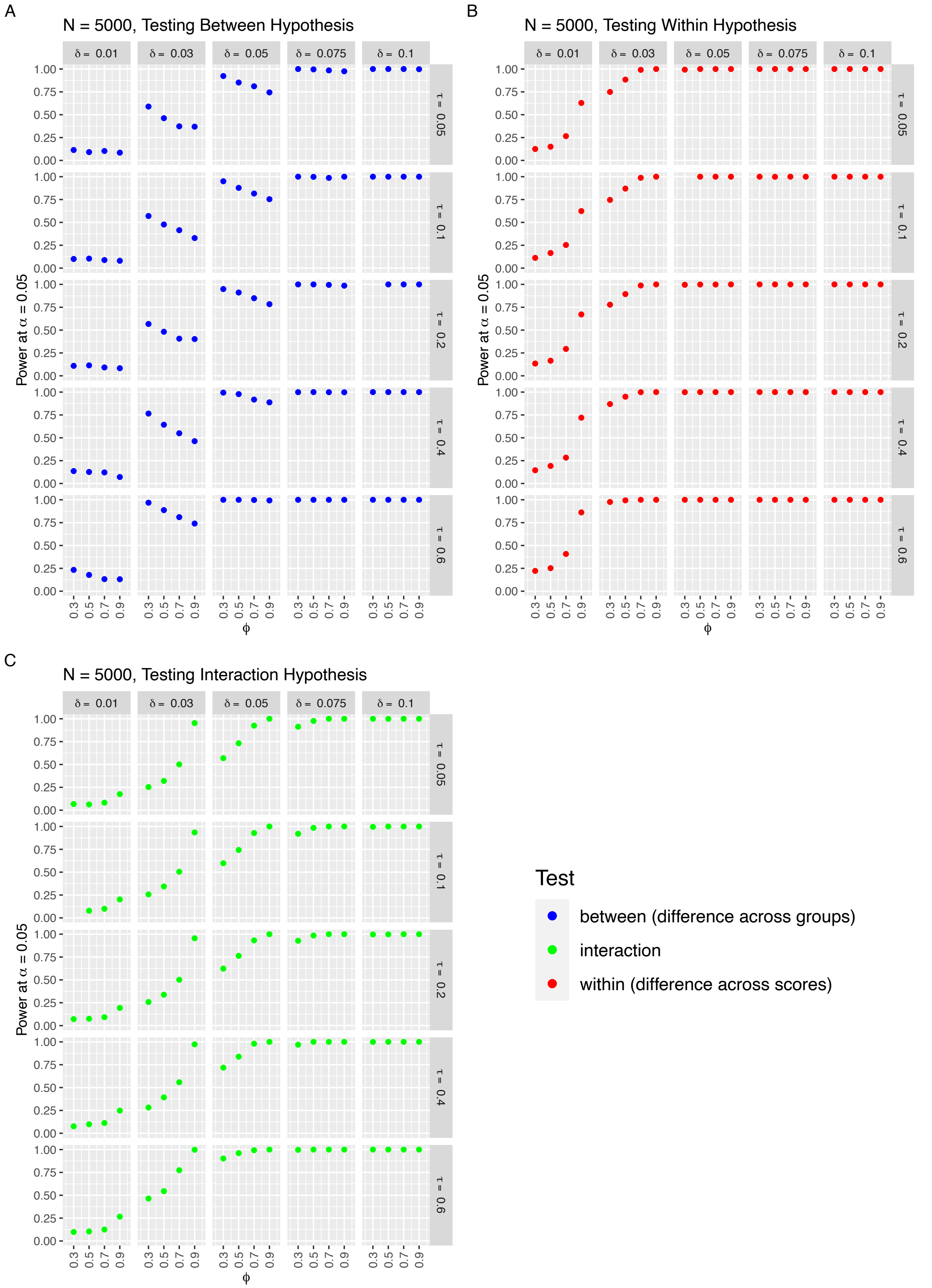

Supplemental Figure E: Power of Parametric Implementation of Coranova with two populations, each of sample size 5000. Each point represents the proportion of tests in 1000 simulations with parameters ϕ, τ and δ in which the null hypothesis was rejected at significance level alpha $= 0.05$.

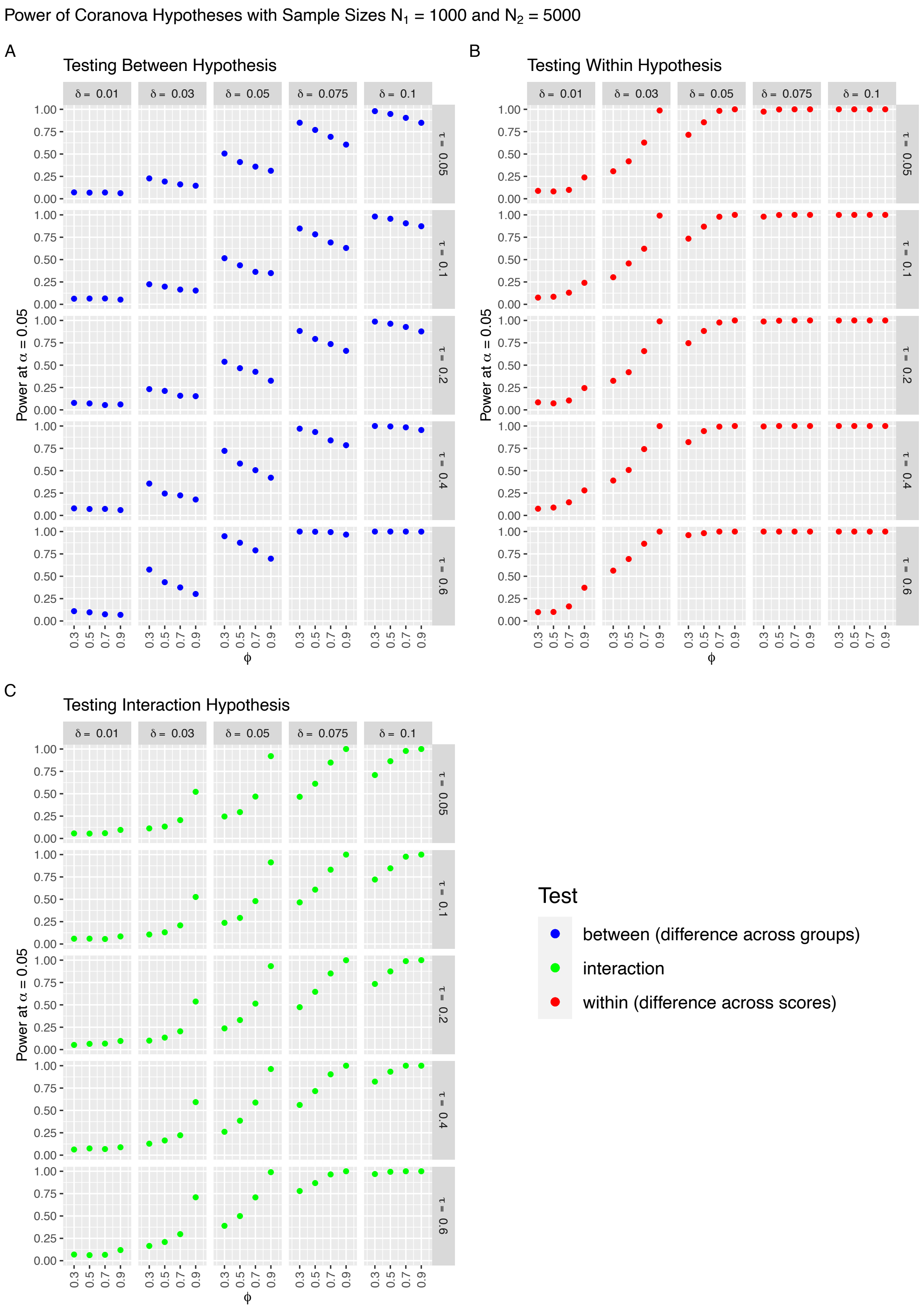

Supplemental Figure F: Power of Parametric Implementation of Coranova with two populations, one of size 1000, one of size 5000. Each point represents the proportion of tests in 1000 simulations with parameters ϕ, τ and δ in which the null hypothesis was rejected at significance level alpha = 0.05.

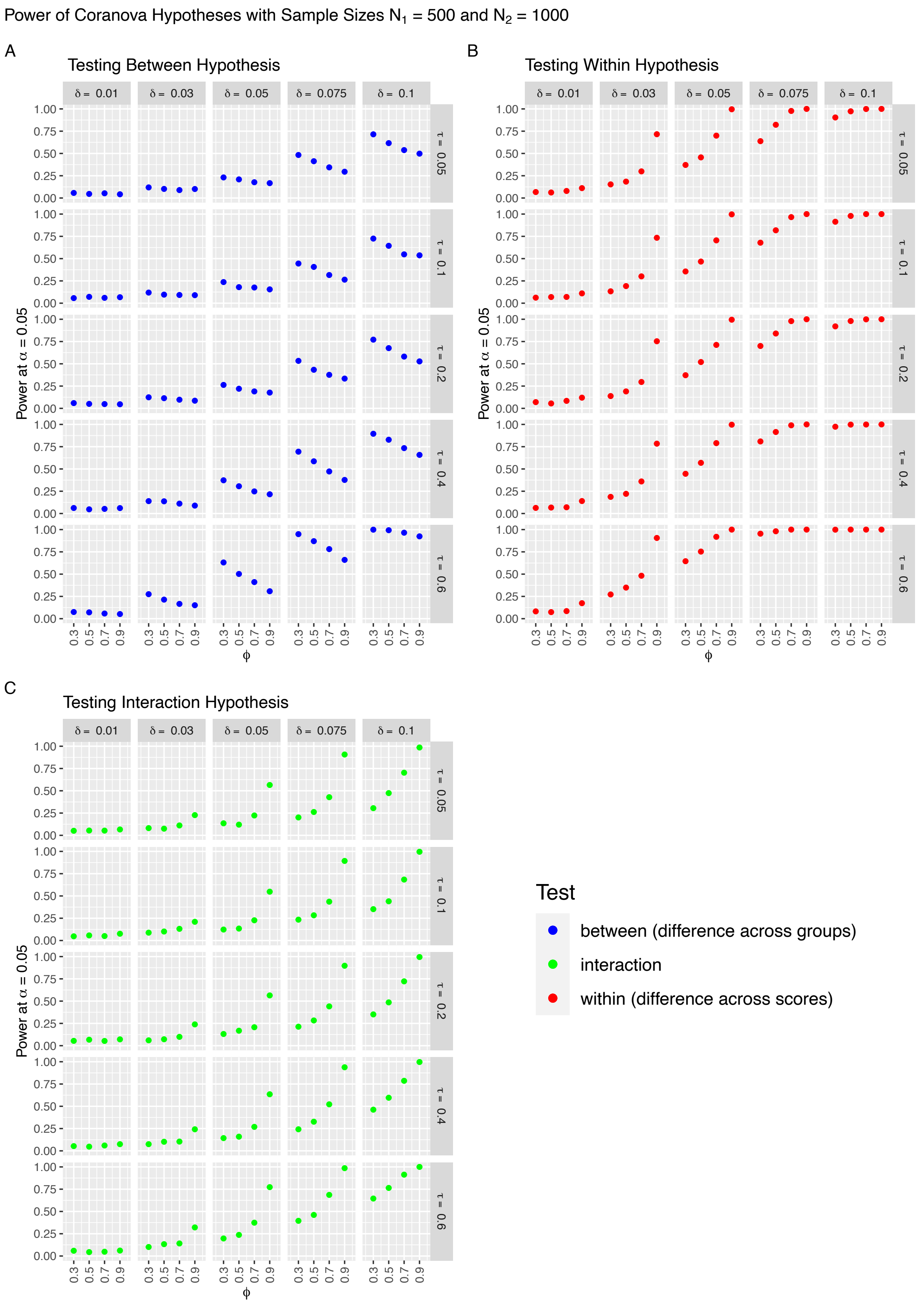

Supplemental Figure G: Power of Parametric Implementation of Coranova with two populations, one of size 500, one of size 1000. Each point represents the proportion of tests in 1000 simulations with parameters ϕ, τ and δ in which the null hypothesis was rejected at significance level alpha $= 0.05$.

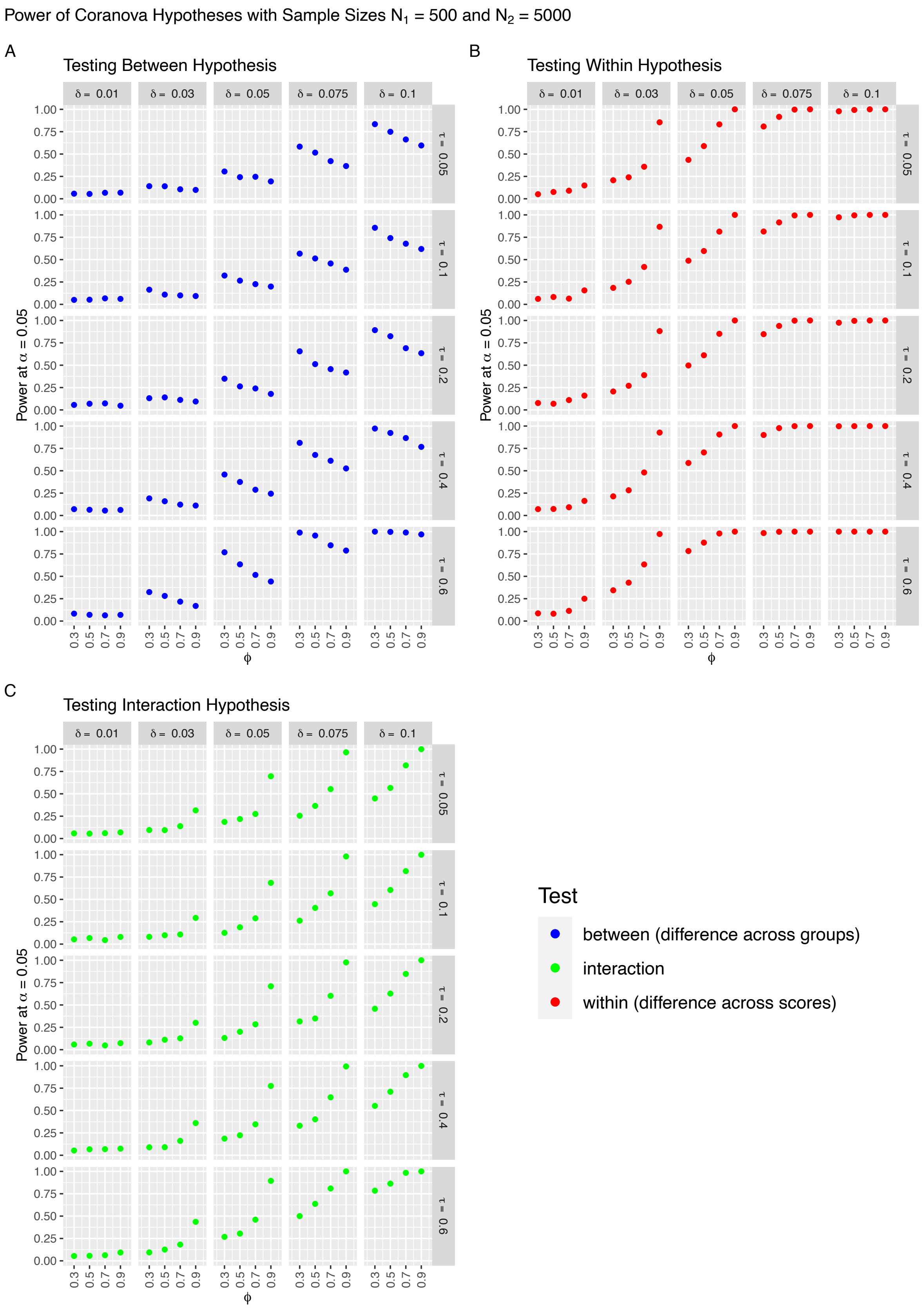

Supplemental Figure H: Power of Parametric Implementation of Coranova with two populations, one of size 500, one of size 5000. Each point represents the proportion of tests in 1000 simulations with parameters ϕ, τ and δin which the null hypothesis was rejected at significance level alpha $= 0.05$.

B Comparison to R2Redux

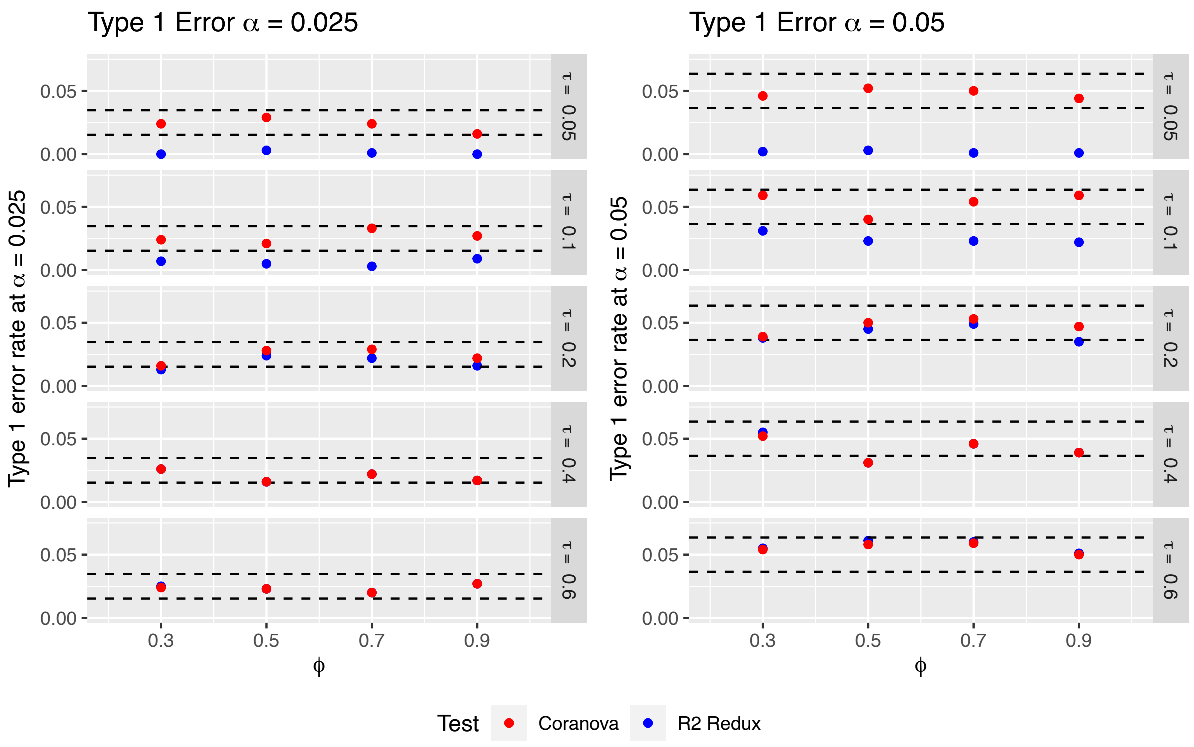

Supplemental Figure I: Type I error for R2 Redux and Coranova methods with two populations, each of sample size 1000 at significance levels alpha = 0.025 and 0.05 for two PGS with equal correlation with outcome Y in a single population sample. Here we present the results of our simulations assessing the type 1 error rate of the two methods when comparing the performance of two polygenic scores in a single population sample, where the two scores are simulated such that they have equal correlation with the outcome $(cor(PGS_1, Y) = cor(PGS_2, Y) = \tau)$. Each point represents the proportion of tests out of 1000 simulations that detected a difference in PGS performance at the specified alpha level for each method under simulation parameters, ϕ and τ. Dashed lines indicate 95% confidence interval for specified alpha given sample size. On the x-axis is correlation between the two PGS, ϕ, and on the rows are the simulation correlation between the polygenic scores and the outcome, τ.

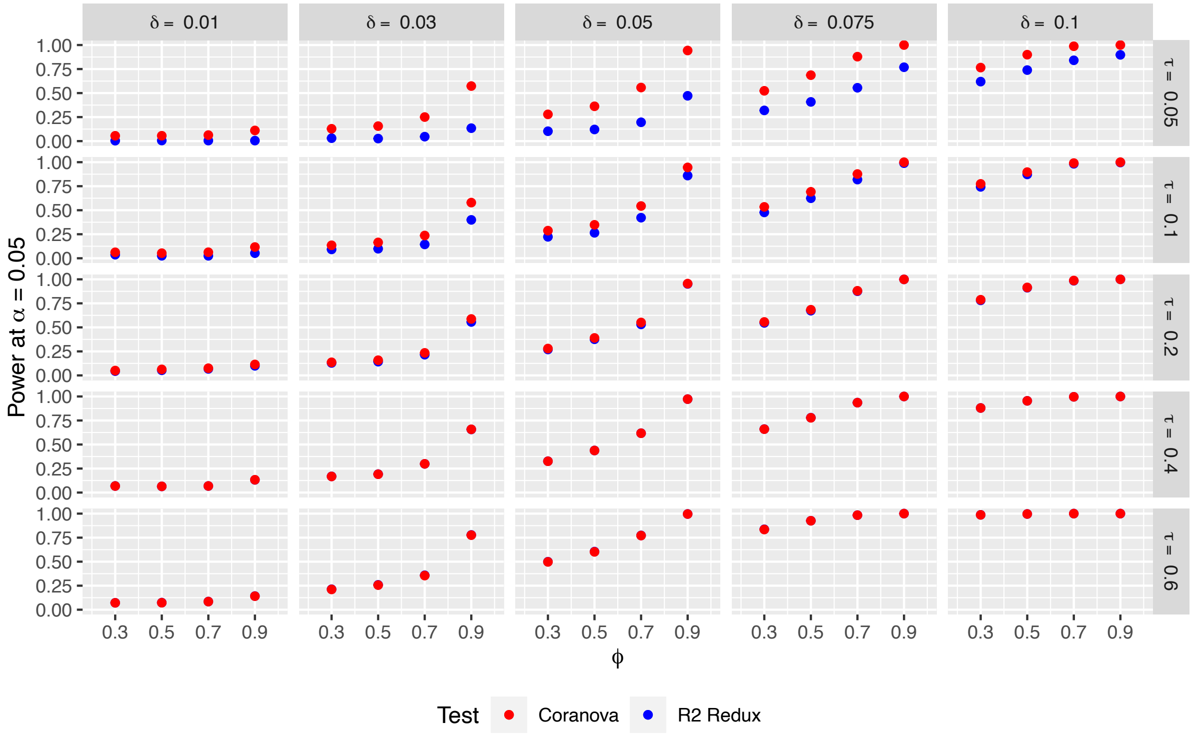

Supplemental Figure J: Power for R2 Redux and Coranova methods with two populations, each of sample size 1000, and significance level alpha=0.05. Here we present the results of our simulations assessing power of the two methods when comparing the performance of two polygenic scores in a single population sample. Each point represents the proportion of tests out of 1000 simulations with parameters ϕ, τ and δ that detected a difference in PGS performance at alpha = 0.05. ϕ is the correlation between the two PGS; τ is the correlation between the first PGS and the outcome, and δ is the difference in correlation with the outcome between the two PGSs.

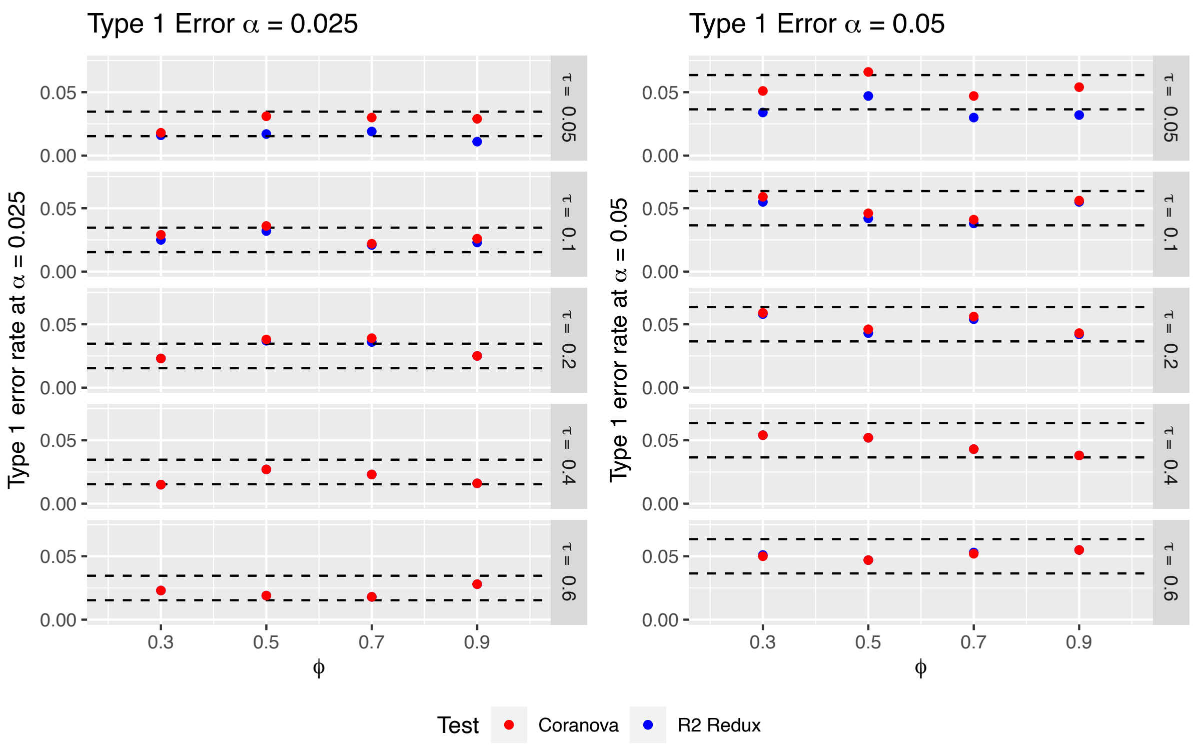

Supplemental Figure K: Type I error for R2 Redux and Coranova methods with two populations, each of sample size 10000 at significance levels alpha = 0.025 and 0.05 for two PGS with equal correlation with outcome Y in a single population sample. Dashed lines indicate 95% confidence interval for specified alpha given sample size.

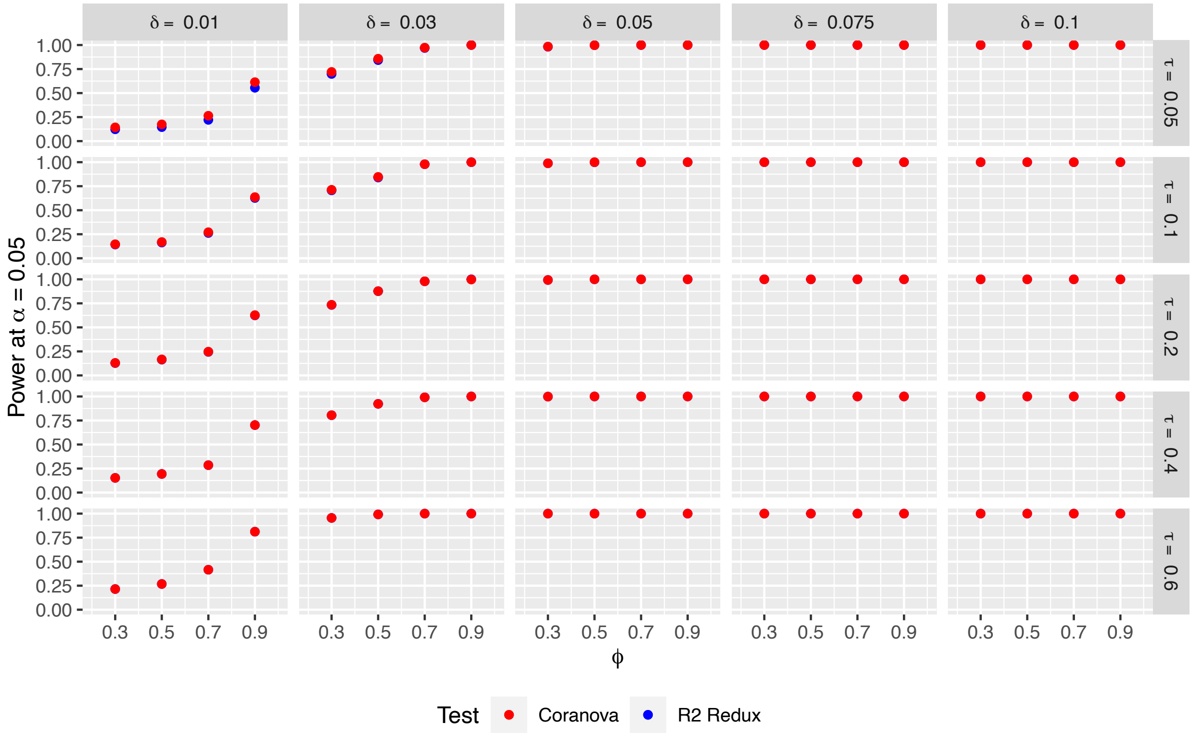

Supplemental Figure L: Power for R2 Redux and Coranova methods with two populations, each of sample size 10000, and significance level alpha=0.05. Each point represents the proportion of tests out of 1000 simulations with parameters phi, tau and delta that detected a difference in PGS performance.

C Nonparametric Implementation Applied to Binary Traits

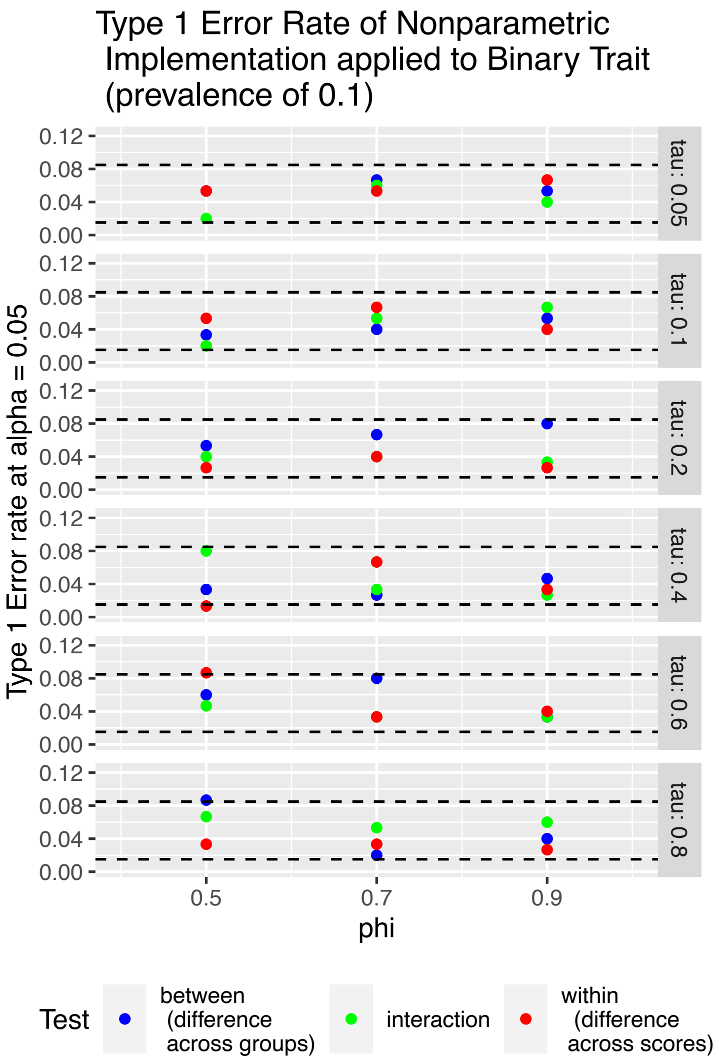

Supplemental Figure M: Type I error of Nonparametric Implementation of Coranova applied to case-control data with two populations of each of sample size 1000, and prevalence of 0.1. Each point represents the proportion of tests in 150 simulations with parameters ϕ, τ and δ in which the null hypothesis was rejected at specified alpha level. Dashed lines indicate 95% confidence interval for specified alpha given sample size.

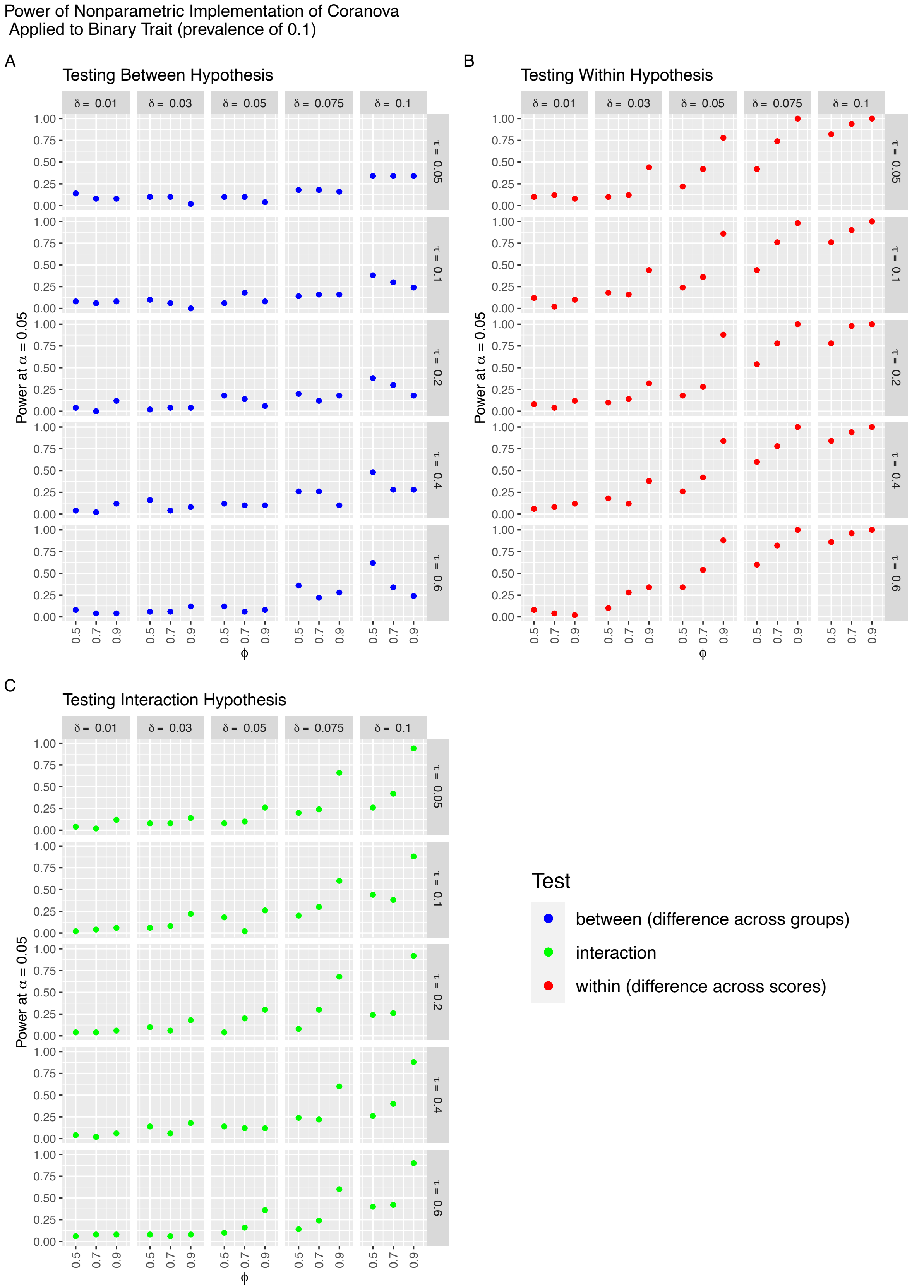

Supplemental Figure N: Power of Nonparametric Implementation of Coranova applied to case-control data with two populations of each of sample size 1000, and prevalence of 0.1. Each point represents the proportion of tests in 150 simulations with parameters ϕ, τ and δ in which the null hypothesis was rejected at significance level alpha = 0.05.
