# Supplementary material for: Correlation-based tests for the formal comparison of polygenic scores in multiple populations
Source: PLoS Genet. 2024 Apr 26;20(4):e1011249. doi: 10.1371/journal.pgen.1011249 (PMC11078427; doi:10.1371/journal.pgen.1011249)
Supplement: S4 Appendix — (PDF) [file pgen.1011249.s004.pdf]

Example 1

```
library(coranova)
library(ggplot2)
```

Example 1: Comparing Three PGS in Two Populations

Say we are interested in comparing the performance of three polygenic scores in an African-ancestry and European-ancestry population, for example in the case of three polygenic scores generated with three different GWAS results: pgs1 from a multi-ancestry meta-analysis, pgs2 from a large European GWAS and pgs3 from a smaller-sized African GWAS. In this case, we are interested in answering a few questions:

Q1. Is the PGS developed from the multi-ancestry meta-analysis better than the PGS developed with ancestry-specific GWASs?

Q2. Does the PGS developed from the multi-ancestry meta-analysis perform better in one population than the other? In this example we will demonstrate how to use our method to address these questions with the coranova command `perform_coranova_parametric` .

With coranova, we are comparing the performance of each PGS alone to predict the outcome using the correlation between the outcome and each PGS as our key metric ($cor(Y, PGS_i)$).

Data Preparation:

To use the coranova package, we need to have one dataframe per population sample where the rows are individuals in the population and the columns include the phenotype of interest, and the polygenic scores we wish to compare.

After loading the package coranova, the two example data frames should be loaded, let's look at the first three rows using the `head` command.

We can see for each population data frame, we have a phenotype vector, and three polygenic scores, named `pgs1`, `pgs2`, and `pgs3`.

```
library(coranova)
head(afr, 3)
```

```
##        pheno       pgs1      pgs2        pgs3
## 1 -1.4606535 -1.1363873  0.798880  0.05945586
## 2 -0.1498208  0.9218904 -1.816460 -1.95811704
## 3  3.5076853  0.2239545  1.586297 -0.12250429
```

```
head(eur, 3)
```

```
##       pheno        pgs1      pgs2       pgs3
## 1 -3.249809  0.66336192 -2.470082 -0.5937651
## 2 -2.754595 -0.72927645  2.431823 -0.1291377
## 3 -1.698634  0.07946734 -1.129588 -0.9772898
```

Let's rename the polygenic score columns to match the GWAS with which they were built:

```
colnames(afr) <- c("pheno", "META", "EUR", "AFR")
colnames(eur) <- c("pheno", "META", "EUR", "AFR")
```

Visualizing Data

To further understand these data, we can also plot the correlations between the three polygenic scores and phenotype within the two populations:

```
library(ggplot2)

cor_dat <- data.frame(pop = c(rep("AFR", 3), rep("EUR", 3)),
                      cor = c(cor(afr)["pheno", c("META", "EUR", "AFR")],
                              cor(eur)["pheno", c("META", "EUR", "AFR")]),
                      score = rep(c("META", "EUR", "AFR"), 2))

ggplot(cor_dat) +
  geom_bar(aes(x = score, y = cor), stat = "identity") +
  facet_wrap(~pop) +
  labs(x = "Polygenic Score", y = "Correlation with Outcome")
```

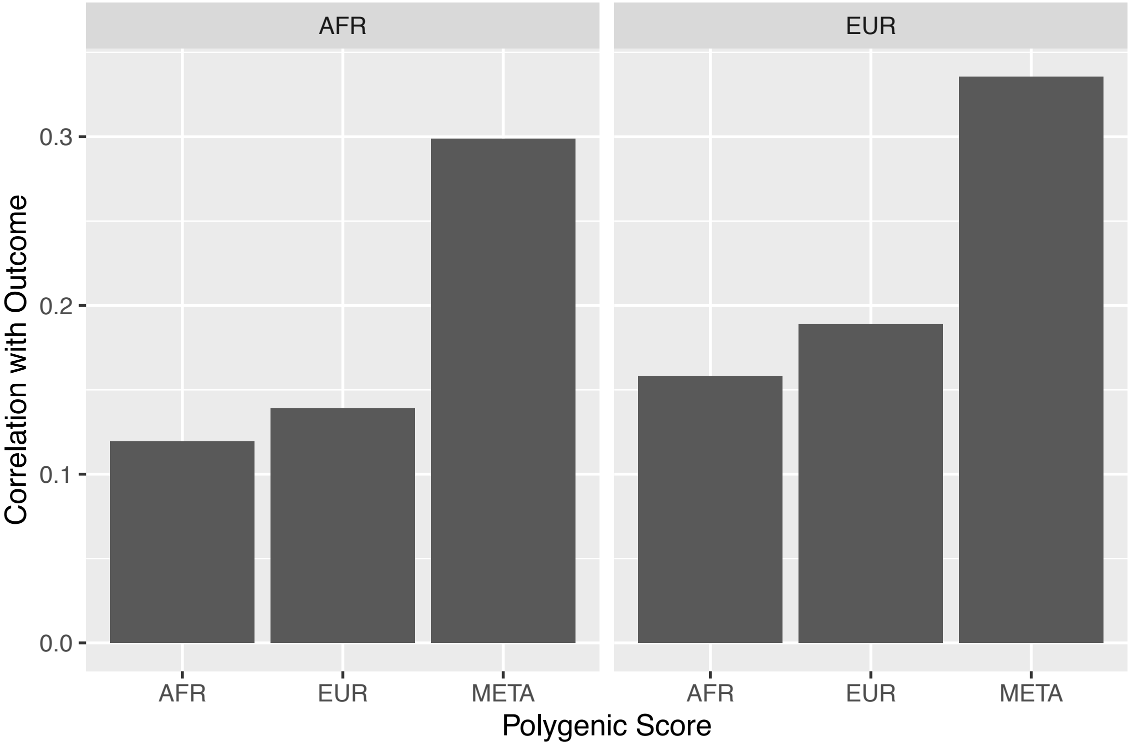

Now, just looking at this figure we can see that the META polygenic score has a much higher correlation with the phenotype in both populations than the other two polygenic scores. However, we want to support

this observation with statistical rigor, which we can do using the coranova package! We can also see that the correlation of the META polygenic score with the phenotype is higher in the European population sample than the African population sample, however we don't know if this difference is statistically significant. We can use this package to determine that!

Using Coranova package

To use the coranova package, we need to wrap the two population sample dataframes in a list with `list(afr, eur)` and provide the column names of the outcome (here `pheno`) and the names of the polygenic scores we want to compare (here: `META`, `EUR`, and `AFR`).

We will run the basic coranova command like this:

```
perform_coranova_parametric(list(afr, eur), "pheno", c("META", "AFR", "EUR"))
```

Comparing Scores across Populations We first may want to establish whether a difference across the three scores within the populations exists, and whether we see a significant difference in score performance across the populations. We can use the basic coranova function `perform_coranova_parametric` to do that.

When we run this:

```
perform_coranova_parametric(list(afr, eur), "pheno", c("META", "AFR", "EUR"))
```

```
## $pB
## [1] 0.0004667585
##
## $pW
## [1] 8.749722e-60
##
## $pI
## [1] 0.8621334
```

The first value `pB` is the p-value from the between test which evaluates whether the mean correlation of the three polygenic scores with the phenotype differs between the population groups `afr` and `eur`. Here the p-value is 0.0005, so we can conclude that the correlation of the scores do vary by population.

The second value `pW` is the p-value from the within test which evaluates whether the polygenic scores have the same correlation with the phenotype or not within the groups. Here the p-value is 9e-60, so we can conclude that the scores do not perform the same in the two population samples.

The third value `pI` is the p-value from the interaction test which evaluates whether there is a difference in the pattern of score performance across the two groups. Here the p-value is 0.86 so we fail to reject the null hypothesis, we can also confirm this visually by noting that in both the `afr` and `eur` population samples, the best performing score is the `META` pgs, followed by the `EUR` pgs, followed by the `AFR` pgs.

Comparing Scores Within Each Population To compare the scores further within each population, we can use `perform_coranova_parametric` with a single population, and the scores we want to compare.

```
perform_coranova_parametric(list(afr), "pheno", c("META", "AFR", "EUR"))
```

If we want to compare the performance of the three polygenic scores within each population, we can use the following commands:

```
## $pW
## [1] 1.574048e-30
```

```
perform_coranova_parametric(list(eur), "pheno", c("META", "AFR", "EUR"))
```

```
## $pW
## [1] 5.36874e-30
```

However, since there are three polygenic scores, the test will just provide the within test p-value (p_{within}). If we want pairwise comparisons, we can run the `perform_coranova_parametric` with a single population and two polygenic scores. However, we recommend using adjusted p-value thresholds, due to the potentially high number of hypothesis tests when comparing pairwise. In this example, we have 2 population samples and three scores, so we will run 2*3 = 6 pairwise tests, thus we will use a p-value threshold of 0.05/6 = 0.0083.

```
perform_coranova_parametric(list(afr), "pheno", c("META", "EUR"))
```

Comparing the pgs1 to the pgs2 to in each population sample:

```
## $pW
## [1] 3.43991e-20
##
## $diff
## [1] 0.1596351
##
## $se
## [1] 0.01734359
##
## $LCB
## [1] 0.1256417
##
## $UCB
## [1] 0.1936286
```

```
perform_coranova_parametric(list(eur), "pheno", c("META", "EUR"))
```

```
## $pW
## [1] 3.037379e-19
##
## $diff
## [1] 0.1469514
##
## $se
## [1] 0.01638739
##
## $LCB
```

```
## [1] 0.1148321
##
## $UCB
## [1] 0.1790707
```

When we only include two polygenic scores, the function returns the difference between the two scores, the standard error of the difference, and its 95% confidence interval.

We conclude that in both population samples, the META has a higher correlation with the outcome than the EUR polygenic score. In the African population sample, the META PGS has 0.16 higher correlation with the outcome than the EUR PGS (95% CI:$(0.12, 0.19), p_{within} = 3.4e^{-20}$). In the European population sample, the META PGS has 0.15 higher correlation with the outcome than the EUR PGS (95% CI:$(0.1, 0.18), p_{within} = 3.0e^{-19}$).

SIDENOTE: We can use the interaction test implemented in coranova to determine whether the difference in performance between the META PGS and EUR PGS differs across the two populations:

```
perform_coranova_parametric(list(afr, eur), "pheno", c("META", "EUR"))
```

```
## $pB
## [1] 0.002737583
##
## $pW
## [1] 8.728204e-38
##
## $pI
## [1] 0.5950258
```

Here, $p_{interaction}$ is 0.59,and thus we conclude that the pattern of score performance does not differ across population samples.

```
perform_coranova_parametric(list(afr), "pheno", c("META", "AFR"))
```

We can also compare the META PGS to the AFR PGS to in each population sample:

```
## $pW
## [1] 3.58535e-24
##
## $diff
## [1] 0.1792939
##
## $se
## [1] 0.01767782
##
## $LCB
## [1] 0.1446454
##
## $UCB
## [1] 0.2139424
```

```r
perform_coranova_parametric(list(eur), "pheno",  c("META", "AFR"))
```

```
## $pW
## [1] 2.333378e-23
##
## $diff
## [1] 0.1773693
##
## $se
## [1] 0.01781223
##
## $LCB
## [1] 0.1424573
##
## $UCB
## [1] 0.2122812
```

We conclude that in both population samples, the META PGS has a higher correlation with the outcome than the AFRP PGS.

```r
perform_coranova_parametric(list(afr), "pheno", c("EUR", "AFR"))
```

Finally, we can also compare the EUR PGS to the AFR PGS to in each population sample:

```
## $pW
## [1] 0.3170777
##
## $diff
## [1] 0.01965877
##
## $se
## [1] 0.01964931
##
## $LCB
## [1] -0.01885389
##
## $UCB
## [1] 0.05817143
```

```r
perform_coranova_parametric(list(eur), "pheno", c("EUR", "AFR"))
```

```
## $pW
## [1] 0.1233748
##
## $diff
## [1] 0.03041785
##
## $se
## [1] 0.01974209
```

```
##
## $LCB
## [1] -0.008276644
##
## $UCB
## [1] 0.06911235
```

Here we do not find a significant difference in performance between the EUR and AFR PGS in either population ($p_{within} = 0.3$ in the African sample, $p_{within} = 0.1$ in the European sample).

Question 4: Comparing META PGS Across Populations Now that we have established that the META PGS performs best in each population, we can compare its performance between populations using the "between" test. If given two populations and a single polygenic score, the function `perform_coranova_parametric` will run this test automatically and provide the difference and 95% confidence interval.

```
perform_coranova_parametric(list(eur, afr), "pheno", "META")
```

```
## $pB
## [1] 0.04006947
##
## $diff
## [1] 0.03692067
##
## $se
## [1] 0.01798349
##
## $LCB
## [1] 0.00167304
##
## $UCB
## [1] 0.07216831
```

When we run this test, we find that the score has a 0.036 higher correlation with the outcome in the European population sample than the African population sample (95% CI: (0.002, 0.072)), and conclude that this difference is significant ($p_{between} = 0.04$).

Conclusions

In this example, we have utilized the coranova package and its command `perform_coranova_parametric` to compare three polygenic scores in two population samples.

We established that the scores' correlation with the outcome did substantially vary within and across populations. We then considered the pairwise differences between the scores to determine than in both populations the `META` PGS has a statistically significantly higher correlation with the outcome than the other two ancestry-specific scores. Finally, we found that the `META` PGS has a slightly higher correlation with the outcome in the European population sample compared to the African population sample, and this difference is statistically significant.

In the second example, we will see how we can use another command in the coranova package `perform_alt_test` to devise more complicated hypothesis tests!

Example 2

```
library(coranova)
library(dplyr)
```

```
##
## Attaching package: 'dplyr'

## The following objects are masked from 'package:stats':
##
##     filter, lag

## The following objects are masked from 'package:base':
##
##     intersect, setdiff, setequal, union
```

```
library(ggplot2)
library(Matrix)
```

In this example, we will demonstrate how to use the function `perform_alt_test` to define and test additional hypotheses!

Some Theory to Set the Scene

First, let's go through a little bit of the math that makes this method possible.

The correlation between two random variables summarizes the strength of their linear relationship, a correlation close to 1 indicates that the two variables have a strong, positive correlation.

In the case of polygenic scores, we would like a PGS to have a high correlation with its associated outcome and we can use correlation to compare PGS.

To derive statistical tests of correlation measures, we can model correlation coefficients as normally distributed, as defined by Olkin & Finn (1990).

Let u be a row vector of sample correlations of p PGS with outcome Y in population j and let μ denote the vector of population values.

$u = (r_{1,j}, r_{2,j}, ...r_{p,j})$
$\mu = (\rho_{1,j}, \rho_{2,j}, ...\rho_{p,j})$

We can model μ as normally distributed with covariance matrix $\hat{\Sigma}(\mu)$, as defined by Olkin & Finn (1990).

$$\mu \sim N(\mu_0, \hat{\Sigma}(\mu))$$

Then, we can test the null hypothesis,

$$H_0 : A\mu = \mu_0$$

where A is a M x p contrast matrix of rank M. Then, Then the test-statistic

$$S = (Au - \mu_0)'(A\hat{\Sigma}_\infty(u)A')^{-1}(Au - \mu_0)$$

will be χ^2 distributed with degrees of freedom M (the rank of contrast matrix A).

For example:

Let $\mu_0 = 0$ and $A^\intercal = \begin{bmatrix} 1 & -1 & 0 & ... & 0 \end{bmatrix}$

Then, $H_0 : \rho_{1,j} = \rho_{2,j}$.

and the null hypothesis is that the first polygenic score has the same correlation with the outcome as the second polygenic score in population j.

Application to PGS

Now let's apply this thinking to an example.

In this example, we will consider 12 PGS prepared using four GWAS bases and three methods (so 4 x 3 total PGS) across two populations.

Let's take a peek at the data:

```
head(pop1, 3)
```

```
##        pheno gwas1_methodA gwas2_methodA gwas3_methodA gwas4_methodA
## 1 -2.0390483    2.24933357     0.8379224     1.9696095     0.9578095
## 2 -1.2930832    0.06856221    -0.6960158    -1.0065659    -1.0096743
## 3 -0.6193357   -0.81564647     0.4009828     0.4275315    -0.1956673
##   gwas1_methodB gwas2_methodB gwas3_methodB gwas4_methodB gwas1_methodC
## 1     1.1287678    0.06747726      2.448551     0.1865982    1.30315265
## 2    -0.2361651   -1.16403068     -1.663347    -0.8044911   -0.01486926
## 3    -0.3996893   -0.19691558     -1.089968    -1.0562738   -0.84349891
##   gwas2_methodC gwas3_methodC gwas4_methodC
## 1   -0.02421069    2.01340288     0.9555608
## 2   -1.59769478   -0.01386361    -1.1787946
## 3   -0.67803075   -0.50727550    -1.1787946
```

```
head(pop2, 3)
```

```
##         pheno gwas1_methodA gwas2_methodA gwas3_methodA gwas4_methodA
## 1 -0.03393565     0.4836638    -1.7878778    -0.5990373     -1.524970
## 2  1.26527908    -0.2655781     0.5214097    -0.7518524     -1.216619
## 3  0.37462066     1.5893040     1.3941387     0.6670144      0.338313
##   gwas1_methodB gwas2_methodB gwas3_methodB gwas4_methodB gwas1_methodC
## 1    -0.9551508    -0.3833416    -0.2438442    0.09568277    -0.5193267
## 2    -0.5338562     1.1358054     2.2409724    2.20115783    -0.9189714
## 3     0.7030845     0.1751785     1.4682204    0.14166627     0.6254887
##   gwas2_methodC gwas3_methodC gwas4_methodC
## 1    -0.5054622     0.7154643     0.5944713
## 2     0.6596678    -0.3319853    -1.1055230
## 3    -0.9456598     1.5870069     1.6388616
```

and look at correlation with the simulated outcome among the scores:

```
cor_dat <- data.frame(pop = c(rep("pop1", 12), rep("pop2",12)),
                      method = rep(c(rep("A", 4), rep("B", 4), rep("C", 4)), 2),
                      cor = c(cor(pop1)["pheno", colnames(pop1)[-1]],
                              cor(pop2)["pheno", colnames(pop2)[-1]]) ,
                      gwas = rep(c("gwas1", "gwas2", "gwas3","gwas4"), 6))
```

In this example, we might be more concerned with comparing methods:

```
cor_dat %>% mutate(score = paste(method, gwas, sep = "_")) %>%
  ggplot() + geom_bar(aes(x = method, y = cor), stat = "identity") +
  facet_grid(gwas~pop) +
  labs(x = "Polygenic Score Method", y = "Correlation with Outcome")
```

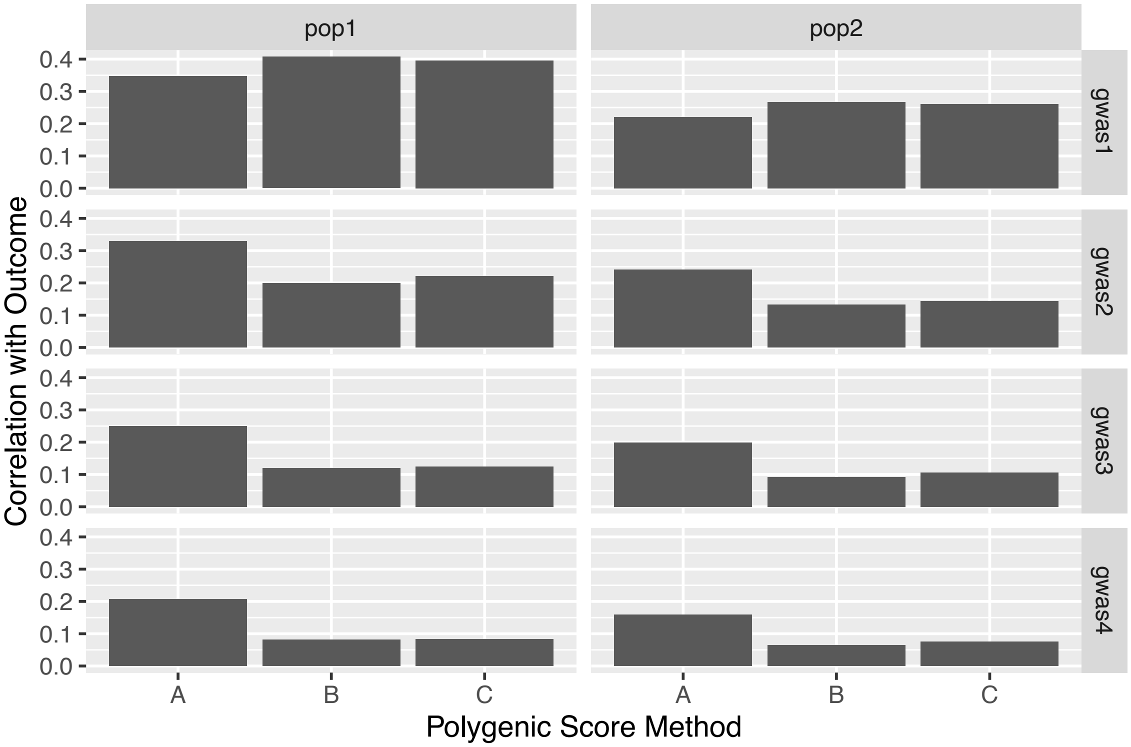

or we might be more concerned with comparing GWAS bases:

```
cor_dat %>% mutate(score = paste(method, gwas, sep = "_")) %>%
  ggplot() + geom_bar(aes(x = gwas, y = cor), stat = "identity") +
  facet_grid(method~pop) +
  labs(x = "Polygenic Score Base GWAS", y = "Correlation with Outcome")
```

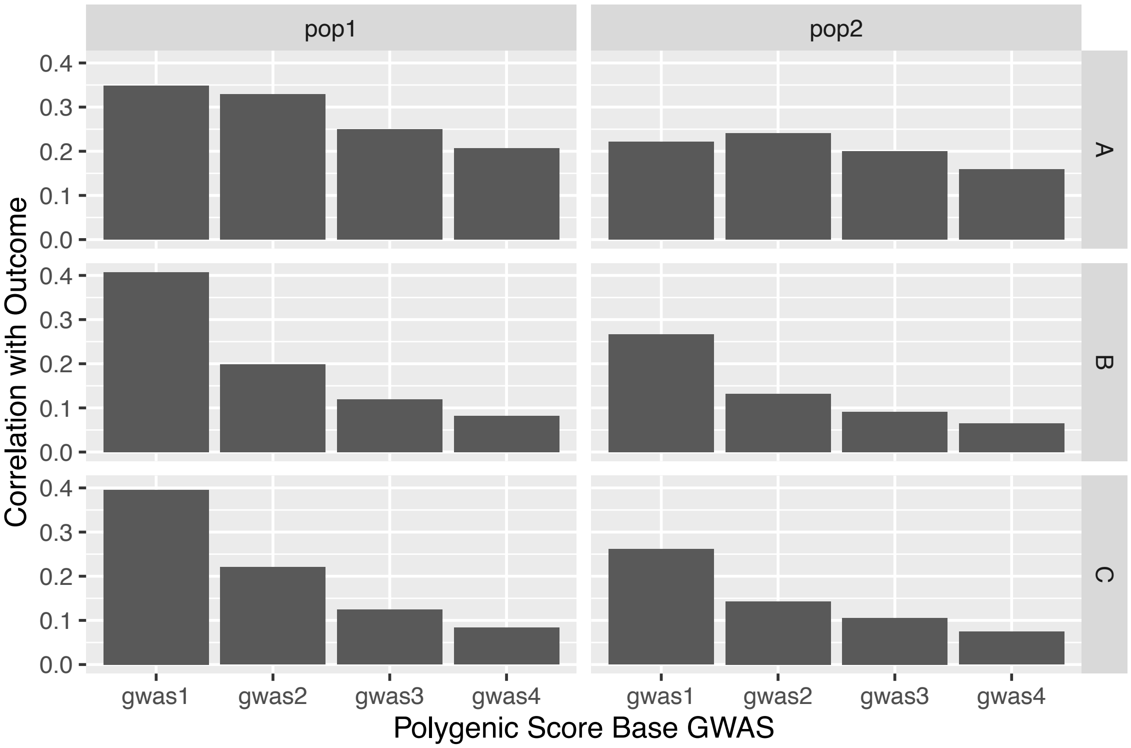

There are many questions we may want to investigate with these data!

```
cor_dat %>% mutate(score = paste(method, gwas, sep = "_")) %>%
  filter(gwas == "gwas1") %>%
  ggplot() + geom_bar(aes(x = method, y = cor), stat = "identity") +
  facet_grid(~pop) +
  labs(x = "Polygenic Score Base GWAS", y = "Correlation with Outcome")
```

Let's start with the small case of comparing the performance of the three methods with gwas1

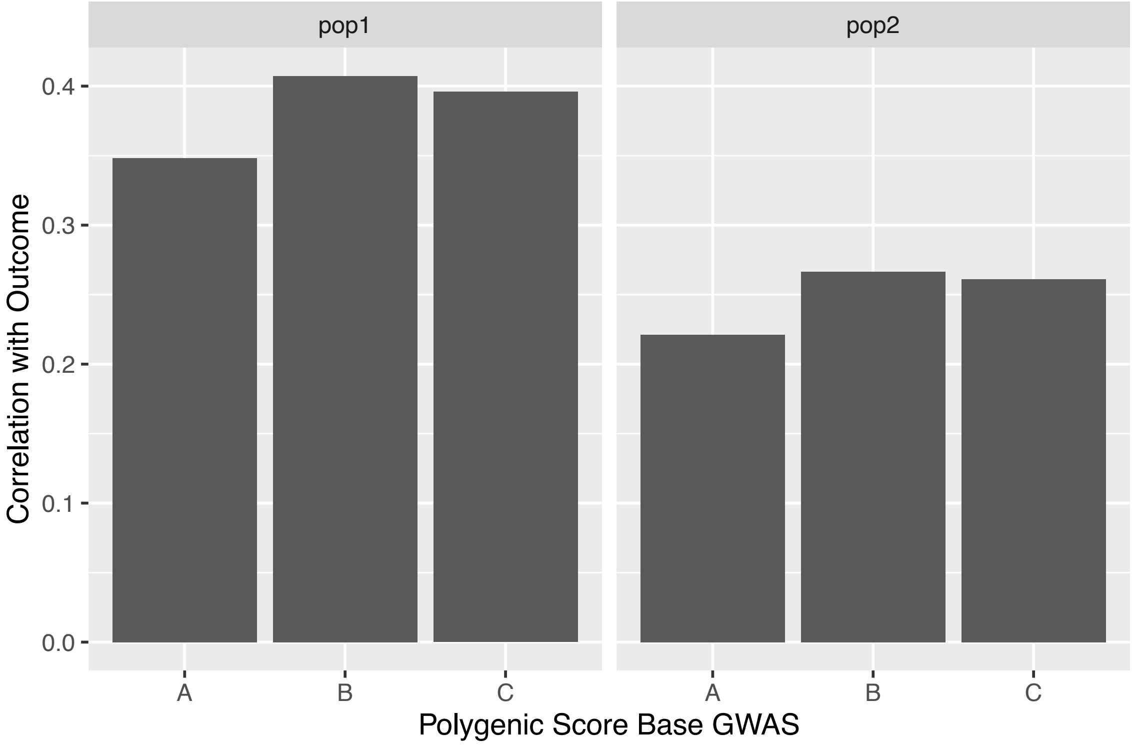

as the base.

We can use the function `perform_alt_test`, to implement this type of hypothesis testing, we just need to specify the:

`dat_list`: list of data frames, where each data frame refers to a separate population sample

`outcome`: name of outcome variable (must be common across dataframes in dat_list)

`measures`: vector of the names of the columns of the measures to be compared

`contrast`: contrast matrix to generate hypothesis test

`method`: to request parametric ("parametric") or bootstrap implementation ("boot") of covariance matrix V

`B`: number of bootstraps if method == "boot" is chosen

In this example, we'll demonstrate how we can define a contrast matrix to determine whether there is a difference in score performance by methods. We will consider only pop1 at first, then show how we can extend this contrast matrix to two populations.

First, let's define the key variables that the function needs:

```
dat_list <- list(pop1)
outcome <- "pheno"
measures <- c("gwas1_methodA", "gwas1_methodB",  "gwas1_methodC")
method <- "parametric"
```

Now, we need to define a contrast matrix to define our null hypothesis to be tested.

Because we have only specified "gwas1_methodA", "gwas1_methodB", and "gwas1_methodC" as our measures, and are only considering pop1, our vector u is:

$$u = (r_{\text{gwas1\_methodA, pop1}}, r_{\text{gwas1\_methodB, pop1}}, r_{\text{gwas1\_methodC, pop1}})$$

if we are interested in comparing the three methods built with the first gwas, we can define a contrast matrix like so

$$A = \begin{bmatrix} 1 & -1 & 0 \\ 1 & 0 & -1 \end{bmatrix}$$

```
contrast <- matrix(c(1, -1, 0,
                     1, 0, -1), nrow = 2, ncol = 3, byrow = T)
```

when we matrix multiply our contrast matrix by our vector of correlations (u),

$$Au = \begin{bmatrix} r_{\text{gwas1\_methodA, pop1}} - r_{\text{gwas1\_methodB, pop1}} \\ r_{\text{gwas1\_methodA, pop1}} - r_{\text{gwas1\_methodC, pop1}} \end{bmatrix}$$

we can see that our null hypothesis is:

$$Au = \begin{bmatrix} r_{\text{gwas1\_methodA, pop1}} - r_{\text{gwas1\_methodB, pop1}} \\ r_{\text{gwas1\_methodA, pop1}} - r_{\text{gwas1\_methodC, pop1}} \end{bmatrix} = \begin{bmatrix} 0 \\ 0 \end{bmatrix}$$

and our alternative hypothesis is that at least one of the pairwise differences (either $r_{\text{gwas1\_methodA, pop1}} - r_{\text{gwas1\_methodB, pop1}}$ or $r_{\text{gwas1\_methodA, pop1}} - r_{\text{gwas1\_methodC, pop1}}$) is not equal to 0 .

we can test this null hypothesis and generate our test-statistic and p-value using the `perform_alt_test` function.

```
perform_alt_test(list(pop1), outcome, measures, contrast, "parametric")
```

```
## $S
##           [,1]
## [1,] 46.43754
##
## $P
## [1] 8.245499e-11
```

And finally, we conclude that at least one of the pairwise differences (either $r_{\text{gwas1\_methodA, pop1}} - r_{\text{gwas1\_methodB, pop1}}$ or $r_{\text{gwas1\_methodA, pop1}} - r_{\text{gwas1\_methodC, pop1}}$) is not equal to 0 ($p = 8e^{-11}$), indicating that there is a difference in the scores generated with the different methods built with gwas1.

To understand how we got to this contrast matrix, let's explain what's going on under the hood of the function. First, we can generate some of the objects used within the function:

```
cormat_list <- lapply(dat_list, cor) #list of correlation matrices, one per
                                     #population sample, here we have just 1 pop,
                                     # so list of size 1
n_list <- lapply(dat_list, nrow) #list of sample sizes, again only 1 pop so,
                                 # so list of size 1
```

To look at the correlation matrix for the polygenic scores we are considering in this example, we can subset like so:

```
cormat_list[[1]][c(outcome, measures), c(outcome, measures)]
```

```
##                   pheno gwas1_methodA gwas1_methodB gwas1_methodC
## pheno         1.0000000     0.3483122     0.4073613     0.3959233
## gwas1_methodA 0.3483122     1.0000000     0.7044181     0.6815255
## gwas1_methodB 0.4073613     0.7044181     1.0000000     0.9690561
## gwas1_methodC 0.3959233     0.6815255     0.9690561     1.0000000
```

Now, let's populate the vector of correlations and their covariance matrix for our test statistic.

```
u <- populate_u(cormat_list, outcome, measures) #u is our vector of the correlations between the polyge
u
```

```
## gwas1_methodA gwas1_methodB gwas1_methodC
##     0.3483122     0.4073613     0.3959233
```

```
V <- populate_sigma(cormat_list, outcome, measures, n_list) #V is the covariance matrix of R, we need t
V
```

```
## 3 x 3 sparse Matrix of class "dsCMatrix"
##
## [1,] 1.544152e-04 9.734042e-05 9.485153e-05
## [2,] 9.734042e-05 1.391301e-04 1.354905e-04
## [3,] 9.485153e-05 1.354905e-04 1.422123e-04
```

```
#we can see that the first term is equal to the correlation of the pgs derived with method A and the ou
# minus the the correlation of the pgs derived with method A and the outcome (R[2])
#and the second term is equal to the correlation of the pgs derived with method A and the outcome (R[1]
# minus the the correlation of the pgs derived with method A and the outcome (R[3])
contrast %*% u
```

```
##             [,1]
## [1,] -0.05904916
## [2,] -0.04761117
```

```
unname(u[1] - u[2])
```

```
## [1] -0.05904916
```

```
unname(u[1] - u[3])
```

```
## [1] -0.04761117
```

Within the function, the test statistic is derived as follows:

```
S <- t(contrast %*% u) %*% solve(contrast %*% V %*% t(contrast)) %*% (contrast %*% u)


p <- pchisq(as.numeric(S), rankMatrix(contrast), lower.tail = F)
```

and this p-value is the probability that our test statistic is at least as extreme as our observed results given the null hypothesis.

```r
S
```

```
## 1 x 1 Matrix of class "dgeMatrix"
##          [,1]
## [1,] 46.43754
```

```r
p
```

```
## [1] 8.245499e-11
```

which is exactly the same as the results of `perform_alt_test`.

```r
perform_alt_test(list(pop1), outcome, measures, contrast, "parametric")
```

```
## $S
##          [,1]
## [1,] 46.43754
##
## $P
## [1] 8.245499e-11
```

That was a lot! But now that we know what we're working with extending to two populations will be no problem! To compare the methods across gwas 1 in both populations,

let

$u = (r_{\text{gwas1\_methodA, pop1}}, r_{\text{gwas1\_methodB, pop1}}, r_{\text{gwas1\_methodC, pop1}}, r_{\text{gwas1\_methodA, pop2}}, r_{\text{gwas1\_methodB, pop2}}, r_{\text{gwas1\_methodC,}}$ [illegible]

we can devise a contrast matrix like so:

$$A = \begin{bmatrix} 1 & -1 & 0 & 1 & -1 & 0 \\ 1 & 0 & -1 & 1 & 0 & -1 \end{bmatrix}$$

Then, our null hypothesis is:

$$Au = \begin{bmatrix} (r_{\text{gwas1\_methodA, pop1}} + r_{\text{gwas1\_methodA, pop2}}) - (r_{\text{gwas1\_methodB, pop1}} + r_{\text{gwas1\_methodB, pop2}}) \\ (r_{\text{gwas1\_methodA, pop1}} + r_{\text{gwas1\_methodA, pop2}}) - (r_{\text{gwas1\_methodC, pop1}} + r_{\text{gwas1\_methodC, pop2}}) \end{bmatrix} = \begin{bmatrix} 0 \\ 0 \end{bmatrix}$$

Essentially here we are averaging across the populations!

Now that we know what we're doing, implementing this is a lot easier. All we need to do is provide the contrast matrix and plug it in to the `perform_alt_test` function. Just make sure you have input the correct population/s, outcome and measures variables!

```r
contrast <- matrix(c(1, -1, 0, 1, -1, 0,
                     1, 0, -1, 1, 0, -1), nrow = 2, ncol = 6, byrow = T)

perform_alt_test(list(pop1, pop2), outcome, measures, contrast, "parametric")
```

```
## $S
##          [,1]
## [1,] 53.63436
##
## $P
## [1] 2.256556e-12
```

Following the same logic, if we want to average across another gwas, all we need to do is change the "measures" option: Here we compare across the scores built with gwas2:

```
contrast <- matrix(c(1, -1, 0, 1, -1, 0,
                     1, 0, -1, 1, 0, -1), nrow = 2, ncol = 6, byrow = T)

measures <- c("gwas2_methodA", "gwas2_methodB",  "gwas2_methodC")
perform_alt_test(list(pop1, pop2), outcome, measures, contrast, "parametric")
```

```
## $S
##          [,1]
## [1,] 140.6314
##
## $P
## [1] 2.899194e-31
```

We can also fully average across all the gwas options and the populations:

$$Au = \begin{bmatrix} \sum_{i=1}^{4}(r_{\text{gwas\_}i\text{\_methodA, pop1}} + r_{\text{gwas\_}i\text{\_methodA, pop2}}) - (r_{\text{gwas\_}i\text{\_methodB, pop1}} + r_{\text{gwas\_}i\text{\_methodB, pop2}}) \\ \sum_{i=1}^{4}(r_{\text{gwas\_}i\text{\_methodA, pop1}} + r_{\text{gwas\_}i\text{\_methodA, pop2}}) - (r_{\text{gwas\_}i\text{\_methodC, pop1}} + r_{\text{gwas\_}i\text{\_methodC, pop2}}) \end{bmatrix} = \begin{bmatrix} 0 \\ 0 \end{bmatrix}$$

```
measures <- c("gwas1_methodA", "gwas1_methodB",  "gwas1_methodC",
              "gwas2_methodA", "gwas2_methodB",  "gwas2_methodC",
              "gwas3_methodA", "gwas3_methodB",  "gwas3_methodC",
              "gwas4_methodA", "gwas4_methodB",  "gwas4_methodC")

contrast <- matrix( c(rep(c(1, -1, 0, 1, -1, 0),4),
                     rep(c(1, 0, -1, 1, 0, -1), 4)), nrow = 2, ncol = 24, byrow = T)

perform_alt_test(list(pop1, pop2), outcome, measures, contrast, "parametric")
```

```
## $S
##          [,1]
## [1,] 130.4708
##
## $P
## [1] 4.662653e-29
```
