# Supplementary material for: Correlation-based tests for the formal comparison of polygenic scores in multiple populations
Source: PLoS Genet. 2024 Apr 26;20(4):e1011249. doi: 10.1371/journal.pgen.1011249 (PMC11078427; doi:10.1371/journal.pgen.1011249)
Supplement: Attachment — Submitted filename: response_to_reviewers.pdf [file pgen.1011249.s005.pdf]

Dear Editorial team and reviewers,

We would like to thank you for your consideration of this manuscript and the thoughtful review of our work. We believe the manuscript has been greatly improved with your comments and suggestions. There are several broad changes we would like to highlight:

1. We have revised most figures and added more complete figure legends.
2. We have expanded our explanation of correlation and why we are utilizing correlation as the basis of our proposed testing framework.
3. We have updated our methods section to be more complete and self-contained, including more fully explaining our simulations framework.
4. We have moved the comparison of our method to R2 Redux to the supplement and revised our discussion of these results to be more complete. We realized it was confusing to have a comparison to R2 Redux be our first set of results. We think that this comparison analysis is more appropriately thought of as a sensitivity analysis to ensure that our correlation-based tests perform as well as R2-based tests in the context of pairwise comparisons of polygenic scores.
5. We have added a brief discussion of application to binary traits and some simulation results.
6. Finally, we have developed two example "vignettes" that now come with our R package. Each vignette takes users through an example of using our functions to compare polygenic scores. The first focuses on the function "perform_coranova_parametric" and will likely be sufficient for the needs of most researchers. The second focuses on the function "perform_alt_test" and leads the user through the process to build more complex hypothesis tests. We have added both vignettes to the supplement pdf submitted as part of this revision.

Below you will find our responses to the specific points raised by reviewers, with the updated text provided.

Thank you very much,
Sophia Gunn and Kathryn Lunetta

Reviewer #1: Correlation-Based Tests for the Formal Comparison of Polygenic Scores in Multiple Populations

Gunn and Lunetta

This study introduces correlation-based methods to assess the performance of Polygenic Scores (PGS) by building upon prior work that developed a statistical framework and robust test statistics for comparing multiple correlation measures across diverse populations. The adaptable framework presented here can be extended to a broader range of hypothesis tests compared to existing methods. The authors validate the proposed methods through simulations and illustrate their utility with two examples: evaluating previously developed PGS for low-density lipoprotein cholesterol and height across multiple populations in the All of Us cohort. Additionally, the authors have created an R package called 'coranova' featuring both parametric and non-parametric implementations of the described methods.

This study is interesting, however, there are comments and questions that should be carefully

considered by the authors.

1. This study employs a correlation-based test as a fundamental aspect of its methodology. It may be essential for the authors to explicitly articulate the distinction between correlation-based and R^2-based tests, as these are two distinct test statistics with unique properties**. I strongly recommend that the authors delve deeper into this comparison, specifically exploring their performance in non-nested model comparisons**. For instance, a valuable approach would be to compare the performance of correlation-based and R^2-based tests using Vuong's test as a benchmark for non-nested model comparisons.

Vuong, Quang H. (1989). "Likelihood Ratio Tests for Model Selection and non-nested Hypotheses" (PDF). Econometrica. 57 (2): 307–333.

For nested model comparisons, a parallel analysis is recommended, with a focus on assessing the performance of correlation-based and R^2-based tests using the likelihood ratio test as a reference. Given the availability of these methods in R-packages, conducting such comparisons should be straightforward.

I think that the R^2-based test primarily serves to assess model fit, akin to the likelihood ratio test for nested model comparisons and Vuong's test for non-nested model comparisons. However, correlation-based test is slightly different from R^2-based or likelihood ratio test including Vuong's.

To enhance clarity for readers, it is advisable for the authors to explicitly elucidate this distinction in the Introduction or early section of the Results, ensuring a clear understanding of the metrics employed in their study.

Thank you for this thoughtful consideration of our work. When we evaluate polygenic scores, we are generally interested in their performance individually, with the goal of selecting the optimal PGS to represent the genetic risk of disease. The model fit tests that you have mentioned are designed to compare models pairwise, determining whether one model fits the data better, or increases the R^2 a significant amount. These tests are very good for pairwise comparisons. The advantage of our proposed methods is that they provide the opportunity for hypothesis tests that are more complicated than a pairwise comparison. Rather, because we can model the vector of correlations of a set of polygenic scores and their associated outcome as normally distributed with a covariance matrix that appropriately models the dependence structure between the correlation values, we can then devise much more complicated hypothesis tests, as long as we can define a linear contrast matrix. Traditional model-fit tests are designed to compare models with potentially many predictors; our proposed correlation-tests only work because we are interested in evaluating the PGS as single predictors.

We have added the following text to the introduction describing the definition of the correlation between two random variables.

Updated Text:
"Correlation measures the linear relationship between two random variables, ranging between -1 and 1. For two random variables X and Y, their correlation is equal to their

covariance divided by their standard deviations ($corr(X,Y) = \frac{cov(X,Y)}{sd(X)sd(Y)}$). In this way, it can be thought of as the normalized covariance between two random variables. In the case of a polygenic score and its associated outcome, this correlation should always be positive, ranging from 0 (the PGS is completely independent of its outcome), to 1 (the PGS is perfectly linearly associated with its outcome). PGS that have higher correlations with their outcomes are better and will be more predictive of their outcomes. The tests we introduce here are designed to compare the correlations of multiple polygenic scores and their associated outcome in multiple populations….

Thus, while the correlation-based tests we introduce here are not the same as R^2-based tests, they will generally lead to the same conclusions as R^2-based tests when performing pair-wise comparisons in samples of size 1000 or larger. Importantly, correlation-based tests also provide the opportunity to test more complex hypotheses about performance of polygenic scores in multiple populations." (pg. 2 lines 80-87, lines 97-101)

We have also added the following to the discussion:

"The methods we propose here are uniquely appropriate for the comparison of performance of polygenic scores. Researchers generally have one of two goals when comparing polygenic scores: 1) selecting an optimal polygenic score for a specific outcome, or 2) determining an optimal polygenic score derivation procedure, comparing scores built with different methods, inputs or both. Both goals often require the comparison of multiple polygenic scores in multiple population samples. Thus, while many methods are available to compare model performance, such as those for nested and non-nested models \cite{vuong1989}, these are not generally appropriate for the needs of researchers studying polygenic scores, as they implement pair-wise comparisons. Our proposed methods can be used to compare many scores at one time, as well as perform pair-wise comparisons as necessary." (pg. 8 lines 355-363)

2. The current study does not address nested model comparisons. Is there a specific rationale for this omission? I recognize that dealing with nested model comparisons can become intricate, especially when multiple predictors are involved. However, it is noteworthy that such comparisons can be readily applied to simpler pairwise scenarios.
Nested model comparison holds significance as it allows for the hypothesis that the inclusion of an additional predictor significantly enhances the model fit. It would be beneficial for the authors to contemplate how this methodology can be effectively employed, particularly when dealing with multiple predictors. Considering and discussing the implications of nested model comparisons in the context of multiple predictors would enhance the completeness and robustness of the study.

Thank you for this insightful recommendation. Tests of nested models enable researchers to understand whether an additional predictor variable improves the fit of the model. The methodology introduced here does not attempt to address that concern because when comparing polygenic scores, we are concerned with which is the best score, not whether the addition of a polygenic score improves the performance of a prediction model which already includes a polygenic score.

The main advantage of this method is that we can devise hypothesis tests to compare multiple polygenic scores at one time, across multiple populations. If a researcher is interested in comparing a model with two polygenic scores to a model with only one, the many nested model tests available should be sufficient.

The text referenced in the previous comment (reviewer #1 comment #1) also addresses this comment.

3. In the abstract, the phrase 'the comparison of multiple correlation measures in multiple populations, perhaps correlated' may be misleading. The term 'correlated' should be omitted, as individuals across populations are assumed to be independent of each other.

Thank you, we have removed the phase "perhaps correlated" from the sentence. It now reads "Here we propose correlation-based methods for evaluating PGS performance by adapting previous work which produced a statistical framework and robust test statistics for the comparison of multiple correlation measures in multiple populations." (pg. 1 lines 8-11).

4. A. The deflated type I error rate is evident in the R2-based test, as illustrated in Figure 1, particularly when tau is low. This observation aligns with expectations, as lower tau values **lead to sign switches in some replicates for the two sets of estimated taus, attributable to sampling errors.** Consequently, the discrepancy between two Polygenic Scores (PGSs) for R^2-based metrics is smaller compared to the correlation-based test. It is noteworthy that, with a larger sample size, the deflated type I error rate tends to be mitigated. To further substantiate this, the authors may consider verification with a substantial sample size, such as n=10,000.

Thank you for this comment and suggestion. We found that only 6.8% of our simulations with a tau of 0.05 lead to a negative correlation, suggesting that sign switching is not the primary factor behind the difference in performance of the two tests. However, we performed simulations with a sample size of 10,000 as suggested and found the tests perform almost identically.

We have revised the main text and included these simulation results in the supplementary materials:

Updated Text:

"Briefly, we also found our correlation-based tests perform very similarly to R2 Redux when assessing the difference in performance between two polygenic scores in a single population (supplement section 1.2.2, and supplemental figures 9 - 12)." (pg. 5 lines 237-239)

Supplement section 1.2.2.
"To compare our correlation-based method to R2Redux, we performed simulations to assess the type 1 error and power of both methods when assessing the difference in performance between two polygenic scores in a single population. We find that our correlation-based method has well controlled type I error in all simulation settings

(supplemental figure 9). With sample sizes of 1000 individuals, we also find our correlation-based method has equal or higher power than R2Redux (supplemental figure 10). When we compare its performance to R2Redux, both methods have well-controlled type I error and equal power when the correlation between the PGS and outcome is high (high τ). When the correlation between the PGS and outcome is low (low τ), the correlation-based tests are more powerful than the tests implemented in R2Redux. However, with larger sample size of 10000, the methods perform nearly identically with respect to type 1 error rate and power (supplemental figures 11 and 12).” (supplement pg. 3-4, lines 651-660).

4. B. Additionally, it is crucial to note that the test outcome depends on the formulated hypothesis. Specifically, the hypothesis should assert no difference between the two sets of R^2 when the direction of association is consistent between the two models. In cases where the direction of association differs, the R^2 for negative regression coefficients should manifest as negative when subjected to subtraction. The authors are encouraged to consider and address this nuance in their analysis.

Thank you for this comment. By definition, a PGS is constructed from GWAS summary statistics in a manner such that higher values of the PGS correspond to higher values of the phenotype, because the weights applied to the genotypes come from summary statistics that associate the variant with the trait. Thus, if a PGS has been properly constructed with the correct effect allele for the beta estimated in the GWAS, the PGS will be positively associated with the outcome. However, a PGS may be negatively correlated with its outcome if the genotypes have been miscoded and the effect alleles are consistently mis-specified (like in the case of confusing the effect and non-effect allele columns). In this case, a simple fix is possible by multiplying the PGS by -1 to ensure that higher PGS values are associated with disease risk. However, a PGS may also be negative if only some of the genotypes are incorrectly coded. We have added a sentence to the introduction to specify a PGS correlation with its associated outcome should be positive and a sentence to our limitations to ensure that researchers know to check for this. We have also added a reminder to check for positive correlation to the user manual available on github.

Updated Text:
Introduction:
“In the case of a polygenic score and its associated outcome, this correlation should always be positive, ranging from 0; the PGS is completely independent of its outcome, to 1; the PGS is perfectly linearly associated with its outcome.” (pg. 2 lines 82-84)

Discussion:
“Finally, the correlation between a polygenic score and its intended outcome should always be positive. If the correlation is not positive, this may indicate an error in designated effect allele in the polygenic score computation. We provide recommendations for implementation of these methods and substantive examples of application in our github user manual.” (pg. 9 lines 441-444)

User manual:
“Prior to running any comparisons, ensure that all correlations between polygenic scores and outcomes are positive. If they are not, the polygenic score was likely miscalculated.

This can happen if the polygenic score was computed with the incorrect effect allele, which in that case it is a simple fix to multiple the polygenic score by -1. However, it is important to check that this is the problem and not a larger problem with the score."

5. In Figure 4, I suggest the authors scrutinize why the test for 'between' decreases as phi increases. Additionally, it would be beneficial to discuss any insights into why the tests for other categories increase with an increasing phi, unless such explanations have already been provided in the text.

Thank you for your suggestion. The reason the between test decreases as phi increases, is because the denominator for the test statistic increases as phi increases. For simplicity, let's consider the case of $i = 2$ polygenic scores and $j = 2$ population samples. Our observations will still hold true in the case of two polygenic scores and three population samples, which is the setting of our simulations. The test-statistic for the between test is as follows:

$$\chi^2_{between} = \frac{\left(r_{11} + r_{12} - (r_{21} + r_{22})\right)^2}{var(r_{11}) + 2cov(r_{11}, r_{12}) + var(r_{12}) + var(r_{21}) + 2cov(r_{21}, r_{22}) + var(r_{22})}$$

Where r_{ij} is the correlation between the ith polygenic score with the designated outcome in the jth population sample. Under our simulation settings,

$r_{11} = r_{21} = \tau$

$r_{21} = r_{22} = \tau + \delta$

$var(r_{11}) = var(r_{21)} = (1 - \tau^2)^2/n$

$var(r_{12}) = var(r_{22)} = (1 - (\tau + \delta)^2)^2/n$

$cov\left(r_{11,} r_{12}\right) = cov\left(r_{12,} r_{12}\right) = (\frac{1}{2}\left(2\phi - \tau(\tau + \delta)\right)(1 - \tau^2 - (\tau + \delta)^2 - \phi^2) + \phi^3)/n$

Holding τ and δ constant, $\chi^2_{between}$ becomes a function of the covariance between the scores $cov(r_{11}, r_{12})$ and $cov(r_{21}, r_{22})$. These covariance terms increase as ϕ increases, and thus the test-statistic $\chi^2_{between}$ decreases.

We do not see this pattern in the within and interaction tests, because those test statistics are different combinations of these terms. In the case of two polygenic scores and two population samples, the within test statistic is as follows:

$$\chi^2_{within} = \frac{\left(r_{11} + r_{21} - (r_{12} + r_{22})\right)^2}{var(r_{11}) - 2cov(r_{11}, r_{12}) + var(r_{12}) + var(r_{21}) - 2cov(r_{21}, r_{22}) + var(r_{22})}$$

Thus, unlike with the between test, holding τ and δ constant, χ^2_{within} increases as ϕ increases, because as ϕ increases, the covariance terms increase and the denominator of the test statistic decreases.

Similarly, in the case of two polygenic scores and two population samples the interaction test statistic is as follows:

$$\chi^2_{interaction} = \frac{(r_{11} - r_{12} - r_{21} + r_{22})^2}{var(r_{11}) - 2cov(r_{11}, r_{12}) + var(r_{12}) + var(r_{21}) - 2cov(r_{21}, r_{22}) + var(r_{22})}$$

The $\chi^2_{interaction}$ test-statistic has the same denominator as the χ^2_{within} test-statistic and therefore, as ϕ increases, the covariance terms increase, the denominator of the test statistic decreases, and the overall interaction test-statistic increases.

We have added the following to the primary text and included an additional supplementary section, “Power as Function of Parameters”.

Main text:
“We also see an increase in power when the PGS are more correlated with the outcome (high τ) due to the inverse relationship with the test-statistics and the variance terms of the correlations (see supplement 1.2.1 for further details).” (pg. 5 lines 219-221)

“We can understand the relationship between power and the correlation between scores by considering the covariance terms in the test-statistics. The between test-statistic ($\chi^2{}_{between}$) is inversely correlated with the covariance terms, while the within and interaction test-statistics (χ^2_{within}, $\chi^2_{interaction}$) are positively correlated with the covariance terms. See supplement 1.2.1 for greater detail.” (pg. 5 lines 226-230)

Supplementary text:

1.2.1 Power as Function of Parameters (supplement pg. 3)

6. Figures 5 and 7 could be significantly enhanced by emphasizing the hypothesis related to multiple PGSs across populations (as depicted in section 3.2). Additionally, it is essential to improve the comprehensibility of the legends associated with these figures.

Thank you for this suggestion. We have updated the figures and figure legends.

Updated figure legend text:
“Figure 3. Correlation of Graham et al. PGS with inverse rank normalized mean LDL cholesterol level in each of the African, Admixed American and European 1KG genetic-similarity groups in AoU. A) The correlation with LDL cholesterol for each PGS by 1KG genetic-similarity group, where each PGS is identified by the GWAS base and method with which it was derived. Sample size of each group specified. B) The average score performance in each AoU 1KG similarity group, corresponding to the "between hypothesis test" in which the null hypothesis is that the scores perform on average the same in the three groups. C) The average score performance in each AoU 1KG similarity group, corresponding to the "within hypothesis test" in which the null hypothesis is that the scores have the same correlation with the outcome when averaged across populations.” (pg. 16)

“Figure 5. Correlation of Yengo et al. PGS with inverse rank normalized mean height in each of the African, Admixed American, East Asian and European 1KG genetic-similarity groups. A) The correlation with height for each PGS by 1KG genetic-similarity group, where each PGS is identified by the GWAS base with which it was derived. Sample size of each group specified. B) The average score performance in each AoU 1KG similarity group, corresponding to the "between hypothesis test" in which the null hypothesis is that the scores perform on average the same in the three groups. C) The average score

performance in each AoU 1KG similarity group, corresponding to the "within hypothesis test" in which the null hypothesis is that the scores have the same correlation with the outcome when averaged across populations.” (pg. 18)

7. When comparing European and African datasets using the GitHub page's function (perform_coranova_parametric(list(afr, eur), "pheno", c("pgs1", "pgs2", "pgs3"))), the author conducted a non-nested model comparison between two independent ancestries, where the sample size can differ between the two populations. Specifically, the author compared the following models:
• Model 1: afr$Pheno ~ afr$pgs1 + afr$pgs2 + afr$pgs3
• Model 2: eur$Pheno ~ eur$pgs1 + eur$pgs2 + eur$pgs3
Is my understanding correct? Nevertheless, it is challenging to discern how the number of Polygenic Scores (PGSs) varies in each population. Additionally, the process may not be very user-friendly, especially if the headers differ across data frames. It is recommended that the authors develop a user-friendly manual and an interface within the functions to enhance usability.

Thank you for this comment. This method uses correlation between each PGS and the outcome in a population sample to assess and compare the performance of polygenic score measures. It is not designed to assess the performance of multiple polygenic scores when combined together in a single model.

We have substantially updated the materials available to users. We have shortened the README available on the initial github page and added two full example vignettes available on github and within R once the package is loaded. We have added the example vignettes to the supplement.

Reviewer #2:
Dr. Gunn and colleagues have introduced a correlation-based approach to test for differences in polygenic scores (PGS) across multiple populations. Utilizing the asymptotic covariance results of correlations from Olkin & Finn, they have developed a test framework and applied it to data from the All of Us project, assessing traits like LDL and height. While the framework is compelling, there are aspects of the manuscript that could be refined for clarity and precision. I suggest the following revisions:

1. On page 3 section 3.1, the definition of \mu as "the vector of population values" should be clarified to represent the population mean of sample correlations. Moreover, the hypothesis testing section should be made more formally precise by presenting the null hypothesis without the term "mean". If u is defined within a specific population j, why don't you define u as u_j=(r_(1,j),…,r_(P,j)) and μ_j=(ρ_(1,j),…,ρ_(P,j)). Then the "between" test null hypothesis can be written as H_0:μ_1=⋯=μ_K. Please update your hypothesis testing section to make it clear.

Thank you for this suggestion. We realize our initial introduction of the parameters was unclear and have updated the Correlation-Based Tests section. The Coranova hypothesis tests we introduce are hypothesis tests based on mean-performance. This is a limitation of the method, and we now address this in the discussion in greater detail.

Updated Text:
"To define our correlation-based tests, we will first define our parameters. Let $\rho_{i,j}$ be the population Pearson correlation of PGS i with outcome Y in population j. Let μ denote the vector of these population correlations of P PGS with outcome Y in population j, $\mu = (\rho_{1,j,}\rho_{1,2}, \dots, \rho_{P,j})$ and let u be the vector of sample estimates of μ, $u = (r_{1,j,}r_{1,2}, \dots, r_{P,j})$." (pg. 3 112-115)

"We also advise caution when performing the between group hypothesis test when comparing many polygenic scores at once, since these tests are designed to detect a difference in mean polygenic score correlation across population groups." (pg. 9 lines 430-433).

2. Figure 1 mentions a parameter \delta that is absent from the figure. The figure should be updated to include this parameter, or the text should be amended to reflect its contents accurately. Additionally, the figure's legend needs more detailed explanations, especially regarding the null hypothesis tested. The same applies to Figure 2, where the legend should provide sufficient context for the results presented.

Thank you for your catch! We have updated the figure caption to remove the mention of delta. We have substantially revised all figure legends. Please also note that the figures 1 and 2 in the original submission are now in the supplemental materials as Supplemental Figures 9 and 10.

Updated Text:
"Supplemental Figure 9. Type I error for R2 Redux and Coranova methods with two populations, each of sample size 1000 at significance levels alpha = 0.025 and 0.05 for two PGS with equal correlation with outcome Y in a single population sample. Here we present

the results of our simulations assessing the type 1 error rate of the two methods when comparing the performance of two polygenic scores in a single population sample, where the two scores are simulated such that they have equal correlation with the outcome (cor(PGS_1, Y) = cor(PGS_2, Y) = τ).Each point represents the proportion of tests out of 1000 simulations that detected a difference in PGS performance at the specified alpha level for each method under simulation parameters, ϕ and τ.On the x-axis is correlation between the two PGS, ϕ, and on the rows are the simulation correlation between the polygenic scores and the outcome, τ." (supplement pg. 13)

"Supplemental Figure 10: Power for R2 Redux and Coranova methods with two populations, each of sample size 1000 at significance levels alpha=0.05. Here we present the results of our simulations assessing power of the two methods when comparing the performance of two polygenic scores in a single population sample. Each point represents the proportion of tests out of 1000 simulations with parameters ϕ, τ and δ that detected a difference in PGS performance at alpha = 0.05. ϕ is the correlation between the two PGS; τ is the correlation between the first PGS and the outcome, and δ is the difference in correlation with the outcome between the two PGSs." (supplement pg. 14)

3. An explanation is needed for why the R2-based test shows significantly less power when the correlation between PGS and outcome is low (e.g., tau = 0.05).

We find that at larger values of N, there is no longer difference in power at low tau levels.

The R2-based tests have less power at lower tau values than the correlation-based tests because of the difference between the test statistics. In the case of two polygenic scores in a single population, the numerator of the correlation-based test-statistic is the difference in the correlation between the two PGS and the outcome ($r_{11} - r_{12}$), where r_{ij} is the correlation between the ith polygenic score with the outcome in the jth population. The numerator of the R2-based test-statistic is the difference in R2 between the two models built with each PGS to predict the outcome. In the case of comparing models with single PGS as the predictor, where the PGS are not very highly correlated with the outcome, the difference in R2 will always be smaller than the difference in correlation because $R^2 = r^2$ and $-1 < r < 1$ ($r > 0$ when the PGS has been properly defined).
At larger correlation values (τ in our simulations) and larger N, the R2 based test performs equivalently to the correlation-based test, but at smaller correlation values and smaller N, this leads to a diminished power of the R2-based test.

Please see our response to reviewer #1 comment #4A for our updated text.

4. Figure 2 is comparing the power of Coranova and R2 Redux. Figure 2 is referenced in the following sentence which focuses on type I error: "We find that our correlation-based method has well controlled type I error when assessing differences in two polygenic scores in a single population (figure 1 and 2)". It would be helpful to describe the results of figure 2 separately.

Thank you for your thoughtful recommendation. We have decided to move the comparisons of R2 Redux and Coranova to the supplement and have also updated the text according to your suggestions.

Updated Text (same as referenced in reviewer #1 comment #4A)

Supplement section 1.2.2.
"To compare our correlation-based method to R2Redux, we performed simulations to assess the type 1 error and power of both methods when assessing the difference in performance between two polygenic scores in a single population. We find that our correlation-based method has well controlled type I error in all simulation settings (supplemental figure 9). With sample sizes of 1000 individuals, we also find our correlation-based method has equal or higher power than R2Redux (supplemental figure 10). When we compare its performance to R2Redux, both methods have well-controlled type I error and equal power when the correlation between the PGS and outcome is high (high τ). When the correlation between the PGS and outcome is low (low τ), the correlation-based tests are more powerful than the tests implemented in R2Redux. However, with larger sample size of 10000, the methods perform nearly identically with respect to type 1 error rate and power (supplemental figures 11 and 12)." (supplement pg. 3, lines 651-660).

5. I am a little bit confused with the setting of Figure 4. Why do you want to conduct the test for "interaction" and "within (difference across scores)" in the setting of "testing between hypothesis"

Thank you for this suggestion. We have altered our figures (fig 2, supp fig 4 – 8) to remove the other tests.

6. Additional clarification is needed for the results depicted in Figure 4, where the power for detecting differences across PGS is higher when the PGS are more correlated with the outcome (high tau). This seems unintuitive, as it suggests that differences should be harder to discern when all PGSs are highly correlated with the outcome.

Thank you for this suggestion. Please see our response to reviewer #1 comment #5, which addresses this concern.

7. The manuscript would benefit from a more detailed description of the simulation settings. Each setting ("between," "within," "interaction") should have clear parameters defined against which the null hypothesis is tested. The current descriptions in the figure legends are brief.

Thank you for this suggestion. We have added greater explanation of the simulation settings after each of the correlation structures are specified.

Updated Text:
Within-test:
"Under this setting, in both populations the third polygenic score has correlation with the outcome Y of $\tau + \delta$ in comparison with the first two polygenic scores which have the same correlation with Y, τ. Thus, when $\delta > 0$,the null hypothesis of equal correlation of the scores within the groups does not hold." (pg. 4, lines 181-183)

Between-test:
"Under this setting, all three polygenic scores in population sample A have correlation τ with the outcome Y, while all three polygenic scores in population sample B have correlation $\tau +$

δ with the outcome Y. Thus, when $\delta > 0$, the null hypothesis of equal correlation of the scores with the outcome across the population does not hold." (pg. 4 lines 188-191)

Interaction-test:
"Under this setting, all three polygenic scores in population sample A have correlation τ with the outcome Y, while the first two polygenic scores in population sample B have correlation τ with the outcome Y and the third polygenic score in population sample B has correlation $\tau + \delta$ with Y. Thus, when $\delta > 0$ the null hypothesis of no difference in pattern of score performance across the groups does not hold, and there is an interaction between the polygenic scores and population samples." (pg. 5 lines 197-201)

8. In the third paragraph of discussion, I am confused with the following sentence: "12 PGS do not have the same correlation with LDLD cholesterol, mean PGS correlation with LDL does not differ by 1KG genetic-similarity group, and the pattern of correlation between the PGS correlation between the PGS and LDL cholesterol differs by genetic similarity group". It would be helpful if you could clarify it a little bit more.

Thank you for your comment. We have amended the text as following:

"In our analysis of the 12 PGS developed by Graham et al. for LDL cholesterol, we apply the three Coranova hypothesis tests and first determine that the 12 PGS do not have the same correlation with LDL cholesterol when averaged across 1KG genetic similarity groups (the "within" hypothesis), the overall the mean of the 12 PGS correlations with LDL does not differ by 1KG genetic-similarity group (the "between" hypothesis), and the pattern of correlations between the 12 PGS and the LDL outcome does not differ across the 1KG genetic similarity groups ("interaction" hypothesis). Between genetic-similarity group and PGS is present. In other words, we find that some PGS perform better than others, but overall, the PGS on average perform the same across the genetic-similarity groups and the pattern of the PGS performance differs by genetic-similarity group." (pg. 8 lines 374-382)

9. The modeling of correlations and the corresponding covariance matrix in your framework shows admirable flexibility, particularly with the potential to use various contrast matrices A for hypothesis testing. The use of a global test to discern differences in the correlation of PRS across ancestries is insightful. However, the true robustness of the approach may be challenged when applied to scenarios involving more than two PRS methods, as is common in practice. In such cases, the global test might encounter limitations due to an increased degrees of freedom penalty, which could impact its power. It would be compelling to see the framework applied to these more complex scenarios. Specifically, it would be valuable if the framework could address detailed questions in a multi-ancestry context, such as:
1) Are there performance differences across ancestries for a given PRS method?
2) Within each ancestry, which PRS method provides the best prediction performance?
3) Considering all ancestries, which PRS method emerges as the most effective?

Thank you for this comment, we have substantially updated the materials available to users. In particular, we have developed the vignette "example 1" to address these proposed questions.

10. When dealing with two PGSs derived from different sources, there might be instances of inverse

correlations with similar magnitudes due to the use of different effect alleles. I'm curious whether the Coranova package can account for such scenarios, which are not typically an issue in R2 evaluations but are pertinent to correlation analyses.

Thank you for this comment. Please see our response to reviewer #1 comment #4B, which addresses this concern.

11. It would be interesting to see an example with more than two PGS methods to demonstrate the method's robustness and applicability.

Thank you for this suggestion! We have provided an example of with 3 PGS methods to our package materials in the vignette "example 2".

12. The supplementary sections 1.1-1.3 could benefit from a more detailed exposition. Some notations are introduced without sufficient background, which could be expounded upon for better clarity.

Thank you for this suggestion, we have updated the sections to introduce all notation (supplement pg. 1).

Reviewer #3: Even though the methods and applications are interesting, the paper needs to be written more carefully and self-contained. Many details still need to be included. Here are my comments, each of which must be addressed.

1. It needs to be clarified how the R2Redux method can address the multiple population context. For a pair of populations, it can be applied. It can possibly be extended for more than two populations. Can the authors clarify this?

As R2Redux is currently described and available, it is only appropriate for the comparison of two model R2 values, or the comparison of a single R2 value to some population value. To our understanding, it cannot be extended for more than two populations, other than by a series of pairwise tests.

2. Methods need to be not written clearly. How does the Coranova model apply in this context? I suggest writing all the details comprehensively. A detailed self-contained methods section is essential. Otherwise, the reader must also read the paper by Bilker et al.

Thank you for this question. We understand the issue to be that we have not sufficiently explained that the Coranova model is a set of hypothesis tests (with their corresponding contrast matrices) that can be employed in the testing framework we discuss in section 3.1 "Correlation-Based Tests." We have updated the "Coranova Hypotheses" section to make it clearer that the Coranova model is a set of three hypothesis tests and their corresponding contrast matrices.

Updated Text:

"Bilker et al. introduced three types of hypothesis tests which enable ANOVA-like testing of correlated variables, like polygenic scores, in multiple population groups by specifying three contrast matrices that can be used in equation (2)." (pg. 3 lines 128-130).

"Let ρi,j be the population correlation between PGS i in population j. We have P PGS and K population samples. The three hypothesis tests can be written as follows;" (pg. 3 lines 138-139)

3. For interaction tests, testing only one pair of PGS ((1,j), (i,j)) may not be sufficient to claim no interaction effects. What about testing the pair ((2,j), (i,j)), and it is rejected? In that case, null is not correct. Please clarify.

The current expression representing the null hypothesis for the Interaction test is correct. In the case of three scores and two groups, the null hypothesis for the interaction test is:

$$(r_{11} - r_{21}) - (r_{12} - r_{22}) = 0$$
$$(r_{11} - r_{31}) - (r_{12} - r_{32}) = 0$$

Where r_{ij} is the correlation of the ith polygenic score with the outcome in the jth population. In its current version, we are comparing the performance of the second and third scores in the two populations to the first score. To demonstrate that it does not matter which score the test compares the others to, we show that we can rearrange these equations to write them in terms of the second polygenic score, by rewriting,

$$(r_{11} - r_{21}) - (r_{12} - r_{22}) = r_{11} - r_{21} - r_{12} + r_{22}$$
$$= r_{11} - r_{12} - (r_{21} - r_{22})$$

And

$$(r_{11} - r_{31}) - (r_{12} - r_{32}) = r_{11} - r_{31} - r_{12} + r_{32}$$
$$= r_{11} - r_{12} - (r_{31} - r_{32})$$

Then, we can see that we can multiply the first equation by -1 to get:

$$r_{21} - r_{22} - (r_{11} - r_{12}) = 0$$

And we can use the fact that under the null $r_{11} - r_{12} = r_{21} - r_{22}$, and rearranging. The second equation we get: $r_{21} - r_{22} - (r_{31} - r_{32}) = 0$, which rearranging gives us:

$$r_{21} - r_{31} - (r_{22} - r_{32}) = 0$$

Thus, the null hypothesis written as:

$$(r_{11} - r_{21}) - (r_{12} - r_{22}) = 0$$
$$(r_{11} - r_{31}) - (r_{12} - r_{32}) = 0$$

Is equivalent to the test written as

$$r_{21} - r_{22} - (r_{11} - r_{12}) = 0$$
$$r_{21} - r_{31} - (r_{22} - r_{32}) = 0$$

Another way to look at this is with matrix algebra:

In the setting of three scores and two populations, the following is the contrast matrix used to derive the test-statistic:

$$\begin{matrix} 1 & -1 & 0 & -1 & 1 & 0 \\ 1 & 0 & -1 & -1 & 0 & 1 \end{matrix}$$

The column vectors of the above matrix are linearly independent.

If we were to also to include a comparison of the second and third polygenic scores, the contrast matrix is no longer linearly independent.

$$\begin{matrix} 1 & -1 & 0 & -1 & 1 & 0 \\ 1 & 0 & -1 & -1 & 0 & 1 \\ 0 & 1 & -1 & 0 & -1 & 1 \end{matrix}$$

This can also be confirmed by reordering the predictor names in the provided R package. The test-statistic associated with the interaction test does not change depending on the order the predictors are analyzed.

4. Simulation study: equal sample size across populations is not realistic.

Thank you for this comment. We have added assessment of type 1 error and power under simulation settings with different population sizes. The results of the assessment of type 1 error are displayed in supplemental figures 3, and the results of the assessment of power are displayed in supplemental figures 6, 7, and 8.

Updated text:

"We simulated population samples of 500, 1000 and 5000, and assessed the performance our methods under equal and unequal sample sizes." (pg. 5 lines 206-207)

5. The simulation design is written ambiguously. It is unclear how different correlation structures are appropriate for testing different hypotheses.

Thank you for this suggestion. We have added greater explanation of the simulation settings after each of the correlation structures are specified. Please see our response to reviewer #2 comment #7.

6. Sampling PGS from a multivariate normal oversimplifies the simulations. It can miss intricate patterns in the performance of the methods. The authors must consider simulating data following the detailed steps of building a PGS.

Thank you for this suggestion. We understand your request to be that we simulate polygenic scores values first by simulating a trait with specific causal SNPs, running a GWAS analysis to return GWAS effect estimates and then generating polygenic scores from the GWAS effect estimates in one or more simulated populations. This type of simulation procedure is appropriate when comparing methods for building polygenic scores because the ground truth of the genetic architecture of the trait is known, and then it is possible to compare how various polygenic score methods perform under certain assumptions of genetic architecture (for example, under certain assumptions of polygenicity or heritability).

We do not believe this type of simulation procedure is appropriate for evaluating the methods proposed here because our methods are not designed to generate polygenic scores, but rather to compare them. For example, in the case of comparing two polygenic scores in a single population (the simplest scenario), the purpose of a simulation study for our methods is to assess under what circumstances, and in in what proportion our method rejects the null hypothesis when the two polygenic scores differ in correlation with the outcome (the method's power), and under what circumstances, and in in what proportion our method rejects the null hypothesis when the two polygenic scores do not differ in correlation with the outcome (the method's type 1 error rate). To accurately assess the power and type 1 error under various parameters, we need to know the ground truth of whether two polygenic scores have the same correlation with the outcome, or by how much they differ in correlation with the outcome to determine whether our method can detect a difference. If we generated two polygenic scores with different methods with the same GWAS, we would not know the ground truth of the difference in correlation between each of the scores and the outcome and thus would not be able to identify whether our method is appropriately rejecting the null hypothesis of no difference or not.

We also believe that using the multivariate normal distribution to simulate the polygenic score values is appropriate, because in practice polygenic scores do follow a normal distribution, and generally researchers scale them to mean 0 and standard deviation 1 within population samples to allow easy comparison of PGS.

7. R^2 and the correlation are the same for normalized response and regressors. Please explain the difference in the type 1 error rate and power study between the two competing methods.

R^2 and correlation are not the same for normalized response and regressors, R2 is equal to the square of the correlation. Thus, when computing the difference between two R2 and two correlations, the values will differ. We recognize that it is not obvious why a test best on the squared correlation would differ in type 1 error and power from that of a test based on the correlation, and indeed, these differences disappear with larger samples. Please see our response to Reviewer 1, comments 1 and 4, where we discuss this in more detail, and describe updates to our manuscript that make this more clear.

8. At least one difference across scores does not indicate which pair of scores has the difference. Authors can discuss subsequent strategies to pinpoint it. Otherwise, we do not know which pair of PGS or populations differ.

Thank you for this suggestion. We have revamped the supporting materials available on github and within the R package to address these concerns. Specifically, the vignette example 1 includes a discussion of how to pinpoint the best performing PGS after determining a difference in score performance among a group of PGS.

9. Instead of rank inverse transformation, what happens if log transformation is applied on LDL and height? Rank inverse transformation should be considered if other primary transformations do not work.

Thank you for this question. Applying a log transformation to LDL and height will yield very similar results. Within the AoU cohort, the correlation between the log of the LDL values and the rank inverse normal transformation of the LDL values is 0.980, and the correlation between the log of the height values and the rank inverse normal transformation of the height values is 0.984.

10. Figure 1 caption: type 1 error should be replaced with type 1 error rate. Y axis label: type 1 error rate. Under null hypothesis, it is not power. There are such mistakes in other parts of the paper as well.

Thank you, we have updated all figures to reflect this suggestion.

11. How does the population structure impact the PGS values itself? Please explain.

Thank you for this question. Population structure largely impacts the polygenic score values themselves via differences in allele frequency. Many variants differ in allele frequency across populations; if such a variant is included in a polygenic score, the difference in allele frequency will lead to a persistent difference in polygenic score values across populations. Problems arise when this difference in allele frequency is correlated with the environment in such a way that influences the trait of interest. We adjust for population structure using principal components of ancestry, to ensure that the relationship between the polygenic score and its outcome is not confounded by population structure.

We have added the following to the main text:

“Additionally, when applying these tests to multiple genetic-similarity populations, we recommend that both outcome and polygenic score are adjusted for genetic principal components prior to analysis. This step is important to ensure that the relationship between the polygenic score and outcome is not confounded by population structure [22].” (pg. 9 lines 432- 435)

22. Alkes L Price, Nick J Patterson, Robert M Plenge, Michael E Weinblatt, Nancy A Shadick, and David Reich. Principal components analysis corrects for stratification in genome-wide association studies. Nature Genetics, 38(8):904–909, July 2006. ISSN 1546-1718. doi: 10.1038/ng1847. URL http://dx.doi.org/10.1038/ng1847.

12. How do the methods perform for disease traits? Please include an analysis.

Thank you for this question! For disease traits, we do not recommend using this method for the comparison of polygenic scores because binary traits cannot be adjusted for principal components of ancestry prior to analysis using a linear model as we recommend for continuous traits. Thus, any analysis of binary traits may be confounded by population structure (see previous comment). However, if this is not a concern for researchers (for example, they are working with a homogeneous population), we have developed a non-parametric implementation of Coranova available in our R package in the function “perform_coranova_nonparametric.” We have updated the text to highlight this function and included some preliminary simulation results of binary traits with prevalence of 0.1.

Updated Text:
Main text:
For this reason, we do not recommend utilizing this method to compare polygenic scores for disease traits, as it is not possible to adjust a binary outcome for covariates with a linear model. If researchers have reason to believe confounding by population structure is not a concern, we have implemented a nonparametric version of Coranova available with the perform_coranova_nonparametric function, which does not assume the data to be normally distributed (see supplemental materials section 1.1.4 and 1.2.3 for more details)." (pg. 9 435-441)

Supplemental Methods:
To simulate binary trait data, we adapted our continuous trait simulations by first simulating a continuous outcome Y and PGS according to the procedure used for continuous traits and then converting the continuous outcome Y to binary by assigning the upper 10% of the distribution to be cases, and the remaining distribution to be controls, generating a binary outcome with prevalence of 10%. We then applied the non-parametric implementation of Coranova available in the function perform_coranova_nonparametric with 1000 bootstrap samples and 1000 permutations. (supplement pg. 1 lines 580 - 585).

Supplemental Results:

We find that with 1000 bootstrap samples, and 1000 permutations, the nonparametric implementations of Coranova have well controlled type 1 error when applied to 3 polygenic scores in 2 population samples of size n = 1000 (supplemental figure 13). Compared to the parametric implementation of Coranova applied to continuous outcomes of equal sample

size, we have diminished power to reject the three Coranova hypotheses when applied to binary data with a prevalence of 0.1 (supplemental figure 14). (supplement pg. 4, 662 – 666).

13. In real data applications, please describe these 12 PGS considered. How were they defined and developed? Everything needs to be written in self-contained manner.

Thank you for this comment. We have updated the paragraph describing the 12 PGS for LDL cholesterol.

Updated Text:
"We considered 12 polygenic scores for LDL cholesterol developed and made publicly available by Graham et al. These PGS were developed by The Global Lipids Genetics Consortium with data from 201 studies. The twelve polygenic scores were optimized using two methods: PRS-CS and pruning and thresholding (PT) with six GWAS performed on samples of varying genetic ancestry, as defined in the original paper. One score for each method is built using multi-ancestry meta-analysis GWAS results and the other five consist of ancestry-specific GWASs of samples from populations of African, East Asian, South Asian, European, and Hispanic ancestry. For the single-ancestry pruning and thresholding scores, a UK Biobank ancestry-matched sample was used to estimate LD, and for the multi-ancestry PT score, a mixed ancestry sample of the UKBB was used to estimate LD. For the single-ancestry PRS-CS scores, the LD reference panels were derived from ancestry-matched samples from 1000 Genomes. A mixed ancestry sample of the 1000 Genomes was used to estimate LD for the multi-ancestry PRS-CS score. The polygenic scores were downloaded from the PGS catalog (publication ID: PGP000230)." (pg. 5 lines 264-275)
