# Supplementary material for: Correlation-based tests for the formal comparison of polygenic scores in multiple populations
Source: PLoS Genet. 2024 Apr 26;20(4):e1011249. doi: 10.1371/journal.pgen.1011249 (PMC11078427; doi:10.1371/journal.pgen.1011249)
Supplement: Attachment — Submitted filename: response_to_reviewers_v2.pdf [file pgen.1011249.s006.pdf]

Dear Editorial team and reviewers,

Thank you very much for your thoughtful review of our work. We have made the suggested changes and responded to the reviewer comments below. After a careful review, we also identified some small typos in the text and an error in two of the figures (incorrect y-intercepts) and have updated the manuscript. We also updated our supplement to clarify the definition of the asymptotic covariance matrix of the sample correlations. We have attached a file which identifies all changes made to the previously submitted manuscript.

Thank you very much,

Sophia Gunn and Kathryn Lunetta

Reviewer #1: In the context of a polygenic score and its associated outcome, the correlation is typically expected to be positive. However, it's essential to note that there can be instances where a negative correlation arises due to factors such as sampling error or a negative association between estimated SNP effects and the outcome.

Please revise 'always' to 'typically' at lines 83 and 441. Other than that, I have no further comments

Thank you for this suggestion. We have updated the language as requested.

The text now reads:

"In the case of a polygenic score and its associated outcome, this correlation is typically positive, ranging from 0 (the PGS is completely independent of its outcome), to 1 (the PGS is perfectly linearly associated with its outcome)." (pg 2, lines 82 – 84)

"Finally, the correlation between a polygenic score and its intended outcome is typically positive. If the correlation is not positive, this may indicate an error in designated effect allele in the polygenic score computation." (pg 9, lines 441 – 443)

Reviewer #2: The authors have fully addressed my comments.

Reviewer #3: The authors have addressed my major concerns. I have the following comments.

1. In the introduction section: .... this correlation should always be positive, ranging from 0 (the PGS is completely independent of
84 its outcome), to 1 (the PGS is perfectly linearly associated with its outcome). This statement requires more justification.

We have updated the language as requested by reviewer #1 to now read, “this correlation is typically positive.”

2. In the responses to the reviewers report, please see the response to comment 3 from the reviewer 3. In the portion where the authors have mentioned contrast matrix using matrix algebra, the columns of the first contrast matrix are not linearly independent. We can add the 1st and second columns to get the last column. Or, did the authors mean the row vectors? Because R2 can not have a basis containing more than 2 linearly independent vectors.

Thank you so much for catching this error! Yes, we meant independent rows. We realized in some places we wrote the test-statistic assuming the contrast matrix was of dimensions p x M in some places and in others where the contrast matrix was of dimensions M x p (where p is the number of polygenic scores and M is the rank of the matrix). We are regretful of this error and have updated the manuscript to ensure that the dimensions of the contrast matrix are consistently m x P, which is consistent with our implementation available on github.
